# Unraveling climate influences on the distribution of the parapatric newts *Lissotriton vulgaris meridionalis* and *L. italicus*

**DOI:** 10.1186/s12983-017-0239-4

**Published:** 2017-12-12

**Authors:** Mattia Iannella, Francesco Cerasoli, Maurizio Biondi

**Affiliations:** Department of Life, Health & Environmental Sciences, University of L’Aquila, Via Vetoio snc Coppito, 67100 L’Aquila, Italy

**Keywords:** Holocene, Last glacial maximum, *Lissotriton*, Multivariate environmental dissimilarity index, Niche divergence, Parapatry, Species distribution models, Sympatry

## Abstract

**Background:**

Climate is often considered as a key ecological factor limiting the capability of expansion of most species and the extent of suitable habitats. In this contribution, we implement Species Distribution Models (SDMs) to study two parapatric amphibians, *Lissotriton vulgaris meridionalis* and *L. italicus*, investigating if and how climate has influenced their present and past (Last Glacial Maximum and Holocene) distributions. A database of 901 GPS presence records was generated for the two newts. SDMs were built through Boosted Regression Trees and Maxent, using the Worldclim bioclimatic variables as predictors.

**Results:**

Precipitation-linked variables and the temperature annual range strongly influence the current occurrence patterns of the two *Lissotriton* species analyzed. The two newts show opposite responses to the most contributing variables, such as BIO7 (temperature annual range), BIO12 (annual precipitation), BIO17 (precipitation of the driest quarter) and BIO19 (precipitation of the coldest quarter). The hypothesis of climate influencing the distributions of these species is also supported by the fact that the co-occurrences within the sympatric area fall in localities characterized by intermediate values of these predictors. Projections to the Last Glacial Maximum and Holocene scenarios provided a coherent representation of climate influences on the past distributions of the target species. Computation of pairwise variables interactions and the discriminant analysis allowed a deeper interpretation of SDMs’ outputs. Further, we propose a multivariate environmental dissimilarity index (MEDI), derived through a transformation of the multivariate environmental similarity surface (MESS), to deal with extrapolation-linked uncertainties in model projections to past climate. Finally, the niche equivalency and niche similarity tests confirmed the link between SDMs outputs and actual differences in the ecological niches of the two species.

**Conclusions:**

The different responses of the two species to climatic factors have significantly contributed to shape their current distribution, through contractions, expansions and shifts over time, allowing to maintain two wide allopatric areas with an area of sympatry in Central Italy. Moreover, our SDMs hindcasting shows many concordances with previous phylogeographic studies carried out on the same species, thus corroborating the scenarios of potential distribution during the Last Glacial Maximum and the Holocene emerging from the models obtained.

**Electronic supplementary material:**

The online version of this article (10.1186/s12983-017-0239-4) contains supplementary material, which is available to authorized users.

## Background

The interpretation of species distribution patterns is crucial to understand biogeographical, ecological and conservation aspects of biodiversity [1–6]. The presence of certain species in a territory can be read as the result of many factors interacting in space and time, such as historical distributions, paleoclimatic events, large and fine-scale fragmentation, biotic interactions, niche width and dispersal ability (e.g., [7–10]). Thus, modelling the distribution of species within discrete biogeographical units, especially when dealing with low-dispersal ability species [11, 12], requires a proper implementation of both Species Distribution Models (SDMs) and phylogeographic analyses based on genetic evidence [13–15]. Species’ distribution is usually constrained by biotic interactions, dispersal capability and geographic accessibility (the “B” and “M” of a BAM diagram [16]) or by abiotic factors (the “A” part). Within the SDMs approach, wide areas with high predicted suitability/probability of presence with respect to a certain species but currently unoccupied suggest the existence of some unmeasured variables constraining the species’ distribution [17]. Dispersal, for instance, could be limited by geographical barriers, competition between two species with similar ecological niches, or tolerance limits to some environmental factors [18]. As an example, cycles of glaciations during the Pleistocene, because of their limiting effects on species’ dispersal possibilities and tolerance to climatic stressors, largely contributed to shape European fauna [19], leading to the extinction of some species and influencing the distribution of other taxa [20–22].

In this contribution, we try to understand if and how the current distributions of the two parapatric newts *Lissotriton vulgaris meridionalis* (Boulenger, 1882) and *L. italicus* (Peracca, 1898), have been influenced by climatic conditions. These two urodeles live in lentic environments, have no strict habitat preferences but differ in some phenological traits [23, 24], and are clearly reproductively isolated [25, 26]. Our analysis is focused on the climatic conditions, particularly those related to temperature and precipitation patterns because of the great influence of these parameters on amphibian life-history traits [27], occurring within the current range of these two amphibians, in order to identify possible variables contributing to limit their capability of range expansion. Particular attention was given to a sympatric zone, because of the great importance accorded to these areas in many fields of theoretical and applied ecology. In fact, understanding the patterns of species’ overlapping occurrences represents a challenging topic of ecological research, ranging from the reconstruction of the species’ evolutionary history [28] to ecological modelling [29, 30], especially when applied to the study of the relationships between past biogeographical patterns and climate in closely related taxa [9, 12, 31]. We built SDMs for both the target species under the current conditions using two machine-learning techniques, Boosted Regression Trees (BRT) and Maxent. Then, the resulting models were also projected to past climatic conditions [21, 32] in order to infer historical habitat suitability and, consequently, to hypothesize how the climatic oscillations during the Last Glacial Maximum and the Holocene have influenced the distribution of these two *Lissotriton* species until they reached the current status. Finally, we investigated also possible statistically significant divergence between the environmental niches of the two target species.

## Material and methods

### Target species and study area

**Fig. 1:**
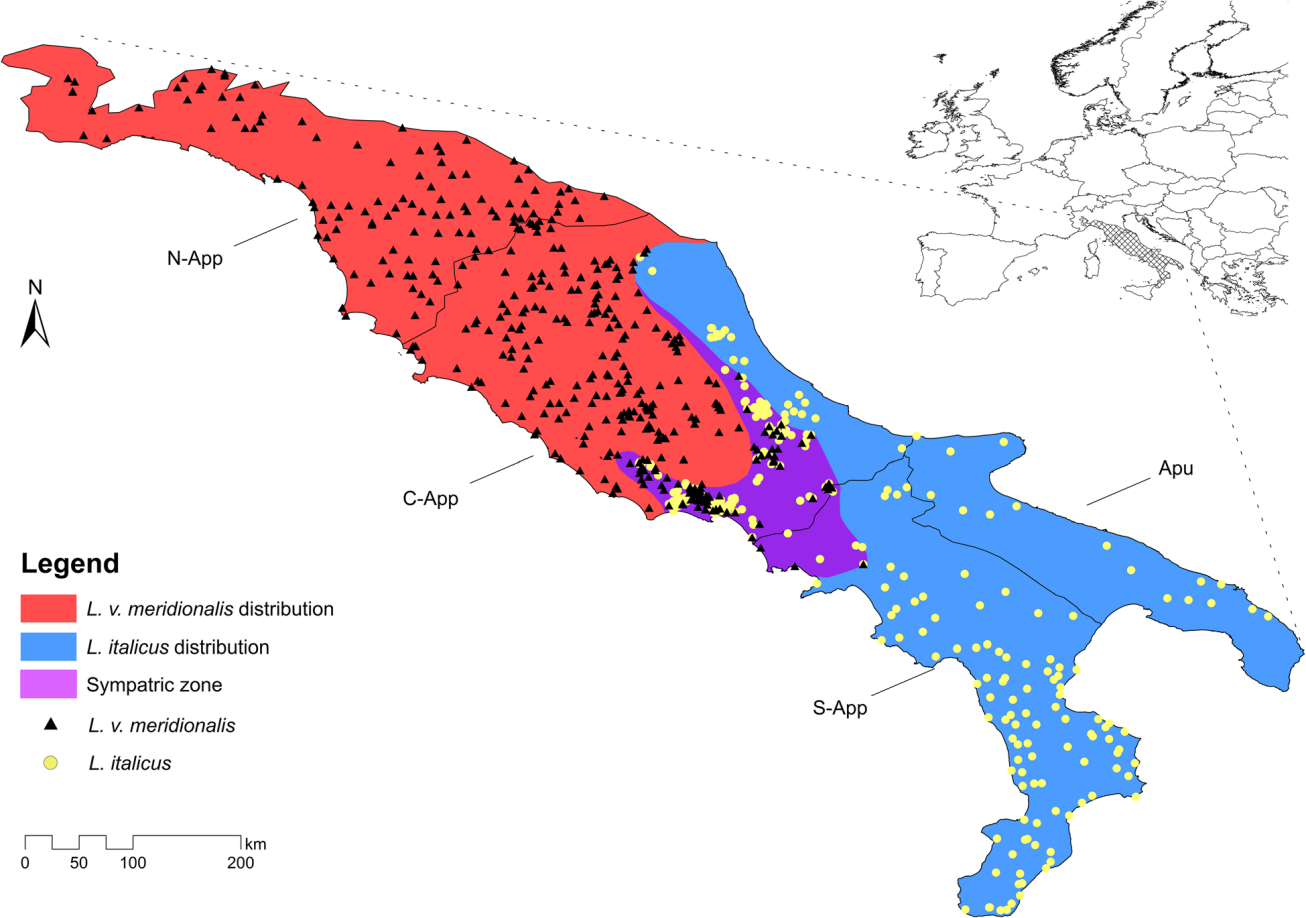
Study area and presence points of the two target species. Study area and distribution of the two *Lissotriton* species, with sympatric area colored in purple. The occurrence records for *L. vulgaris meridionalis* are shown as black triangles while those for *L. italicus* as yellow circles. N-App: Northern Apennines, C-App: Central Apennines, S-App: Southern Apennines and Apu: Apulian Province, modified from Minelli et al. [85] and Biondi et al. [86]

Two amphibians of Caudata, *Lissotriton italicus* (Italian newt) and *L. vulgaris meridionalis* (Southern smooth newt), were considered in our analysis. These newts are endemic (*L. italicus*) or sub-endemic (*L. v. meridionalis*) to peninsular Italy [33], where they show a widely complementary distribution with an overlapping area in Central Apennines (Fig. 1).

Presence records in the study area were drawn from the database reported in Iannella [34], with a total of 401 and 500 occurrences for *L. italicus* and *L. v. meridionalis*, respectively; 25 records, all falling into the sympatric area, are referred to syntopic localities shared by both species. The geographic coordinates of the presence records are reported in the Additional file 1.

Our study area comprehends the whole Apennine area and the Apulian Province, since the latter hosts two *L. italicus* haplogroups shared with some other *L. italicus* populations of Central and Southern Apennines [35]. We did not consider, however, the portion of *L. v. meridionalis’* range corresponding to the Padano-Venetian plain (sensu Canestrelli et al. [36]) and to the Prealps, since our aim was to focus on the climatic conditions of the Mediterranean biogeographical region where the parapatry between our two target species occurs. Moreover, we excluded the *L. v. meridionalis’* populations located in north-eastern Italy because of the introgression events with *L. v. graecus’* populations from northern Balkans [37]. However, since restrictions of the environmental range in SDMs may lead to artefacts in response curves and spurious projections [38], we investigated if the set of environmental conditions characterizing the portion of the *L. v. meridionalis*’ range not included in our study area was noticeably different from the one used to calibrate our model. 170 presence and 1000 background points were randomly sampled from each of the two portions of *L. v. meridionalis’* range (included and excluded); the values of the input bioclimatic variables at each point were extracted using the ‘Extract Values to Points’ tool in Arcmap 10.0 and the Pearson coefficient (*r*) between the resulting matrices was computed in R through the ‘cor.test’ function. Presence records for *L. v. meridionalis* outside the study area considered for this correlation test were drawn from [34].

### Model building and GIS analysis

In order to build the SDMs for the target species, Boosted Regression Trees (BRT) and Maxent were used. Both are among the modelling techniques which perform best, based on statistics referring to discrimination, calibration and correlation between observations and predictions [38, 39]. Contrarily to the high number of contributions based on Maxent analyses [40], there are not as many studies implying BRT to address biogeographical questions, probably because it requires a more substantial effort to parameterize and build the model. Nevertheless, BRT was chosen because this technique permits to accurately model complex responses of the target species to the predictors [39, 41], and to investigate possible synergistic effects of the considered variables through the assessment of pairwise interactions. The BRT models were built in R 3.3.2 (R Core Team, 2016) using the package ‘gbm’ 2.1.1 [42] and the ‘gbm.step’ function provided by J. Elith and J.R. Leathwick [41], while the Maxent models were built through the software MAXENT 3.3.3 k [43]. Notwithstanding BRT is a “tolerant” method with respect to correlated variables [44], a correlation matrix considering all the variables selected as possible predictors was built (see Additional file 2), in order to exclude the ones showing Pearson | *r* | > 0.85 [38], thus avoiding any multicollinearity side effect. Bioclimatic variables of current, Mid-Holocene (MOL, ~ 6000 years ago), and Last Glacial Maximum (LGM, ~ 22,000 years ago) periods and three topographic variables were considered as candidate predictors. Nineteen bioclimatic variables were downloaded from the WorldClim.org website, version 1.4 [45], at 30 arc-seconds resolution, when available. LGM bioclimatic variables, available only at 2.5 min resolution, were rescaled to 30 arc-seconds in ArcMap 10.0 [46]. For both the Mid-Holocene and the Last Glacial Maximum, two Global Climate Models (GCMs) were considered, namely the CCSM4 [47] and the MIROC-ESM [48]. The details on the bioclimatic variables are reported in Additional file 3. With regard to the topographic variables, altitude (ALT) was obtained through the digital elevation model of the study area, downloaded from the geo-portal of the Italian Ministry of the Environment (http://www.pcn.minambiente.it). The other two topographic variables, aspect (ASPECT) and slope (SLOPE) were derived from ALT through their respective functions in ArcMap 10.0.

A two-step pseudo-absence selection method with preliminary environmental profiling was adopted [49, 50] to generate the pseudo-absences used to build the BRT models. The environmental profiling was performed by building a Bioclim [51] model in R through the package ‘dismo’ 1.1–1 [52], for each species, considering as predictors the bioclimatic variables which resulted to be non-correlated from the above-mentioned correlation matrix. Bioclim continuous output was converted in five discrete classes in GIS environment (ArcMap, Natural breaks) and the pseudo-absences were generated by selecting points at random within the polygons corresponding to the three classes of lowest modelled habitat suitability, so as to characterize the pseudo-absences as possible true absences. The use of pseudo-absences generated after a prior identification of climatically unsuitable areas allowed us to properly model the potential distribution of the two *Lissotriton* species considered [49, 53]. The number of the pseudo-absences generated for each species through this procedure was the same as the number of presence records, since a ratio between presences and pseudo-absences close to one seems to assure an optimal performance of BRT models [54]. The BRT models were built with the following setting of parameters for each species: 10-fold cross-validation; ‘tree complexity’ = 5; ‘bag fraction’ = 0.5 and ‘learning rate’ = 0.001. Maxent, instead, was parameterized as follows: ‘Auto features’; 10-fold cross-validation; 10,000 background points; 5000 iterations. Additional SDMs were built for the current scenario, through both BRT and Maxent, considering as predictors both the non-correlated bioclimatic variables and the topographic ones, in order to assess if the latter outclass the climate-linked variables in terms of contribution to the models.

In order to point out possible spatial autocorrelation of presence data before building the SDMs [55], a Moran’s I test on a 5 × 5 km grid, derived from the 10 × 10 km UTM grid, was performed for each species, with each cell containing spatial information of presence data. The raster values used in the statistical analysis were extracted through the ‘Extract Values to Points’ tool in ArcMap 10.0.

All spatial input data (i.e. presence records and rasters of the candidate predictors) were clipped to the extent of the whole study area and projected in WGS84 reference system.

### Model evaluation

BRT and Maxent models obtained were evaluated in their discrimination power by means of the AUC (i.e. Area Under Curve) of the ROC (Receiver Operating Characteristic) curve [56], a threshold-independent statistic which assesses the capability of a SDM to discriminate between presences and (pseudo-)absences [38, 43]. Secondly, calibration within the BRT models was evaluated considering the deviance score, calculated through the ‘gbm.step’ function, which represents a measure of loss in predictive performance due to suboptimal models [41]. Both for the BRT and the Maxent models, the relative contribution of each predictor was assessed, and the partial dependence plots for the most contributing ones were successively produced. Moreover, within the BRT models, the pairwise interaction score of each possible pair of predictors was computed in order to individuate any synergistic effect between the most influential bioclimatic variables. Finally, a forward stepwise discriminant function analysis [57] was performed to derive functions discriminating the respective allopatric and sympatric areas for *L. italicus* and *L. v. meridionalis*, using a selected group of predictors. No data standardization or normalization were performed for these variables.

Statistical analyses and graphics were performed using the package NCSS version 11 for Windows.

### Model projection to past scenarios

SDMs obtained for the two target species under the current conditions were projected to the LGM and MOL scenarios using both the CCSM4 and the MIROC-ESM paleoclimatic reconstructions. A multivariate environmental similarity surface (MESS) [58] was computed in order to quantify the degree of extrapolation (i.e. prediction to environmental conditions that differ from those represented within the training data [55]) that the obtained SDMs would require when projected to the considered past scenarios [59]. This analysis was performed using the ‘mess’ function implemented in the ‘dismo’ package. For each combination of species-scenario-GCM, the percent extent of the study area showing MESS values lower than −20 was calculated in Arcmap 10.0.

SDMs’ projections resulting from the two different GCMs were combined firstly by averaging them (‘Mean’ consensus approach [60]); then, in order to reduce the influence of possible spurious projections due to model extrapolation [58, 60, 61], a weighted average was also computed by assigning to the projection resulting from a GCM a weight being inversely proportional to the degree of “environmental novelty” of the GCM’s hindcasted climate with respect to the current conditions across the presence-background points. In this way, the more the SDM projection considering a certain GCM requires extrapolation, the less it contributes to the combined projection, so that this latter would be driven mostly by the projections having less intrinsic uncertainty.

In order to plainly formulate the above-mentioned weighted average (i.e. avoiding negative weights which could drive the values of the averaged projection outside the range 0–1), a transformation of the multivariate environmental similarity (MES) [58] to a positively-valued multivariate environmental dissimilarity index (MEDI) is here proposed.

MEDI value at each point is derived from the MES value at that point as follows:

$$ \mathrm{MEDI}\kern0.5em =\kern0.5em 100\kern0.5em -\kern0.5em \mathrm{MES}. $$

Hence, MEDI values higher than 100 (corresponding to MES < 0) will indicate sites with one or more variables outside the range of values characterizing the reference points (i.e. non-analog climate [62]), while MEDI values lower than 100 (MES > 0) will indicate sites with environmental conditions within the range of the reference points, and increasingly common across the reference sites as MEDI tends to 0.

Thus, our weighted average (Proj_WA_) of the SDM projections (Proj) for a certain species (Sp) to a past scenario (Sc) considering *n* GCMs will be:

$$ {\mathrm{Proj}}_{\mathrm{WASpSc}}=\frac{\sum_{i=1}^n{\left(\frac{1}{{\mathrm{MEDI}}_{GCMi}}\right)}^{\ast }\ {\mathrm{Proj}}_{GCMi}\ }{\sum_{i=1}^n\left(\frac{1}{{\mathrm{MEDI}}_{GCMi}}\right)} $$

We propose the use of this weighted average, based on the inverse of MEDI, because it is concurrently intuitive and proper with respect to the aim of down-weighting extrapolation. Indeed, for example, in case we attributed weights based on the inverse squared MES values, as a common transformation to avoid negative weights, analogue and non-analogue environments with the same MES absolute value (but with positive and negative sign, respectively) would have the same weight.

### Niche divergence

To test for possible statistically significant differences between the environmental niches of the target species, two different methods were used. The first one is based on the comparison of the observed value of niche overlap, estimated by calculating the Schoener’s *D* metric [63] on SDMs-derived geographical projections of the suitability/probability of occurrence for the two target species, to a null distribution of overlap *D* values computed on simulated niches built through randomization procedures [64–66]. This approach was implemented through the ENMTools software [67]. The second method is based on a preliminary analysis by means of an ordination technique aimed at reducing the set of input variables to two principal component axes defining a gridded environmental space inside which the kernel-smoothed density of occurrence of the two target species is calculated taking into account the density of the different combinations of environmental conditions available to each species [64]. Also in this case, the observed overlap *D* value between the two species in the gridded environmental space is compared to a null distribution of *D* values computed between simulated niches built through randomization procedures [64]. For this latter method the R package ‘ecospat’ [68], using the ‘PCA-env’ [64] as ordination technique, was used.

For both the approaches, we first tested for niche equivalency and then extended the analysis to the less restrictive hypothesis of niche similarity [65], comparing the observed values of niche overlap to the 95th percentile density of the simulated values forming the null distribution [64, 65]. The random points used to perform the background test in ENMTools were generated through the Sampling Design Tool in ArcMap 10.0 [69].

The *ecospat.plot.niche.dyn* function was used to create plots of each species’ occurrence density with respect to the single input variables [68].

## Results

The 77.6% (388) of the 500 occurrences analyzed for *Lissotriton v. meridionalis* falls in the allopatric area, while for *L. italicus* the percentage is only of 43.1% (173/401). The sympatric area, located in Central and part of Southern Apennines, corresponds to the 16.4% of the total area of *L. italicus*, with 228 occurrences, and to the 14.8% of the total area of *L. v. meridionalis*, with 112 occurrences (Fig. 1).

Presence data showed no spatial autocorrelation for both species (Moran’s *I* = 0.186, *p*-value = 0.348, z-score = 0.939 for *L. italicus* and Moran’s *I* = 0.161, p-value = 0.426, z-score = 0.796 for *L. v. meridionalis*).

On the basis of the obtained correlation matrix (see Additional file 2), we selected eleven out of the nineteen Worldclim bioclimatic variables, namely BIO2, BIO4, BIO5, BIO6, BIO7, BIO8, BIO9, BIO12, BIO15, BIO17 and BIO19.

The correlation analysis between the matrices representing environmental conditions within the portions of *L. v. meridionalis’* range falling inside and outside the study area resulted in *r* = 0.990 (*p* < 0.001). Thus, it can be reasonably assumed that the SDMs built for *L. v. meridionalis* on the considered study area are properly informed about the full set of climatic conditions relevant to the species, so that the Padano-Venetian plain and Prealps can be excluded from the study area without weakening model calibration.

The following results refer primarily to the models built through the BRT technique. However, we have also reported in an additional table and in an additional figure (see Additional file 4) the results of the Maxent analyses, which were performed in order to verify the overall consensus of predictions between different model classes [21]. Both the techniques provided very similar responses with respect to the two target species considered.

Models of current distribution obtained through the BRT technique showed high discrimination for both species, with a cross-validated AUC = 0.892 ± 0.008 for *L. italicus* and a cross-validated AUC = 0.854 ± 0.011 for *L. v. meridionalis*. Models built through Maxent achieved similar, though lower, scores, with cross-validated AUC = 0.851 ± 0.009 for *L. italicus* and cross-validated AUC = 0.794 ± 0.013 for *L. v. meridionalis*. With respect to the calibration performance shown by the BRT models, as measured by the deviance scores, similar patterns emerged for both *Lissotriton* species: the model built for *L. italicus* showed an initial mean total deviance of 1.386, and the final model, resulting from 12,100 trees, yielded a mean residual deviance of 0.468; for *L. v. meridionalis*, instead, starting from the same mean total deviance of 1.386, the final model, built on 10,050 trees, yielded a mean residual deviance of 0.615.

**Fig. 2:**
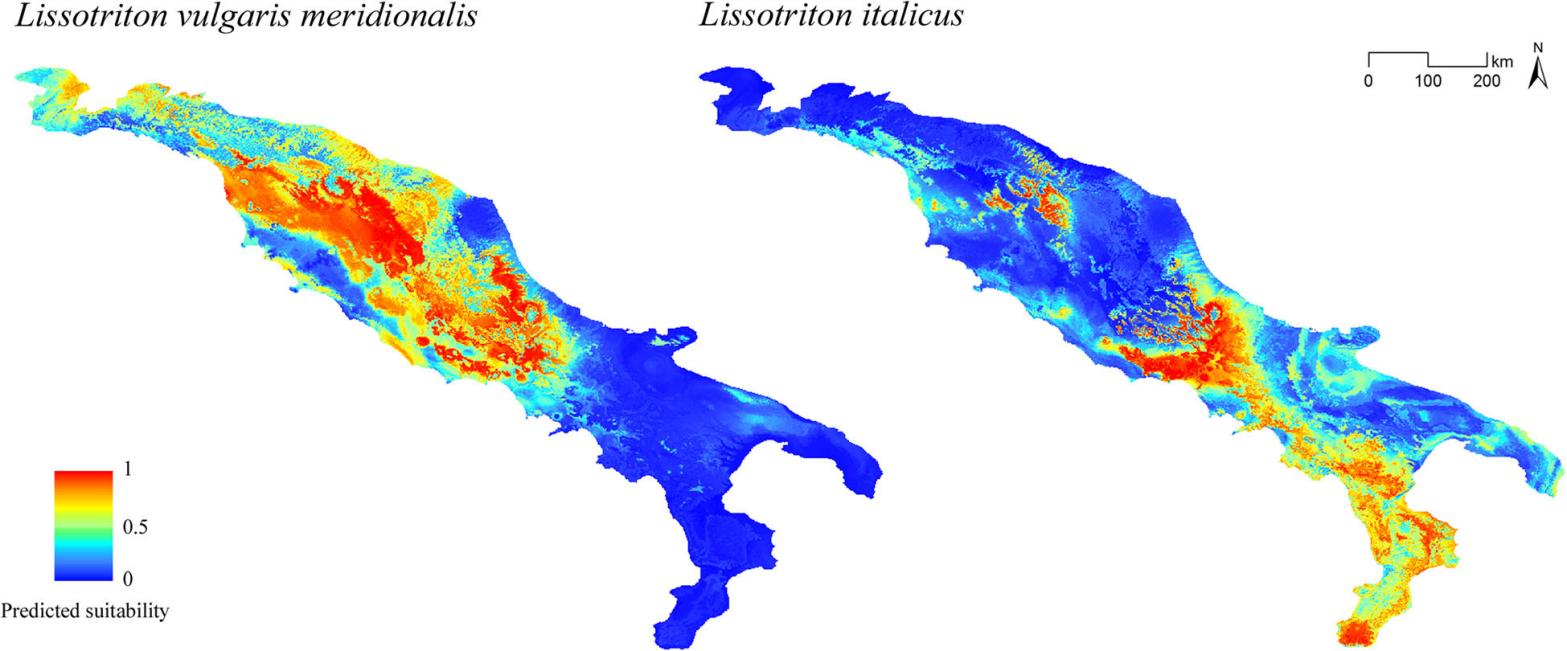
Modelled distribution of *Lissotriton vulgaris meridionalis* and *L. italicus* under current climatic conditions. Maps representing the modelled distribution of *Lissotriton vulgaris meridionalis* and *L. italicus*, as resulted from the corresponding cross-validated BRT models based on the current bioclimatic conditions

Maps resulting from the BRT models for *L. v. meridionalis* and *L. italicus* on the current bioclimatic conditions are shown in Fig. 2, with the predicted suitability reported in the continuous format. By comparing the two maps, it emerges the existence of a portion of Central Apennines with favorable bioclimatic conditions for both *Lissotriton* species.

As evidenced by the cross-validated BRT models obtained, precipitation of the driest quarter (BIO17) and precipitation of the coldest quarter (BIO19) were found to be the most influential variables for *L. v. meridionalis* and *L. italicus*, respectively. The six most contributing variables and the relative percentages of contribution are reported, for each species, in Table 1.

**Table 1:** Relative contribution of the six most influential predictors for each target species

*Lissotriton italicus*		*Lissotriton vulgaris meridionalis*	
Variable	Relative contribution (%)	Variable	Relative contribution (%)
BIO19	17.7	BIO17	22.3
BIO12	12.7	BIO2	11.8
BIO17	12.0	BIO12	10.2
BIO7	10.4	BIO4	10.1
BIO4	10.1	BIO8	8.3
BIO9	7.5	BIO7	7.2

For each *Lissotriton* species are reported the six most contributing predictors and their relative contributions, as results from the corresponding cross-validated BRT models

**Fig. 3:**
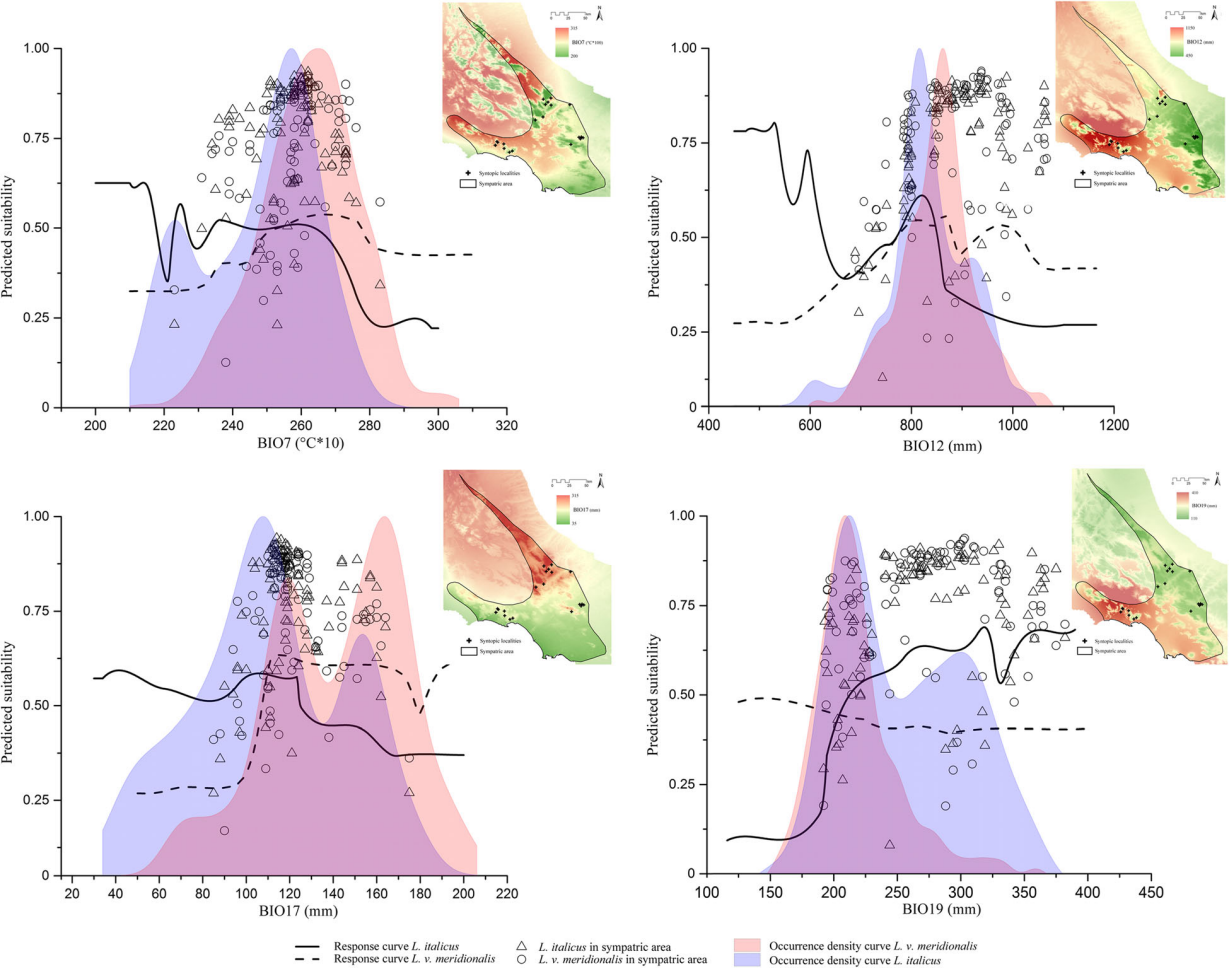
Marginal response and occurrence density curves of four highly contributing predictors common to both species. Response curves of the four predictors included among the six most contributing ones for both *Lissotriton italicus* and *L. vulgaris meridionalis* within the cross-validated BRT models built on the current bioclimatic conditions. Predicted suitability for *L. italicus* (triangles) and *L. v. meridionalis* (circles) in the occurrence localities within the sympatric area is shown on each plot, together with the respective occurrence density curves obtained through the Ecospat package. A map showing the values of the corresponding predictor within the portion of the Central Apennines including the sympatric area is also shown beside each plot

The response curves in Fig. 3 show the trends of BIO7 (temperature annual range), BIO17, BIO12 (annual precipitation) and BIO19 for both the *Lissotriton* species. Along with the response curves, in each plot are shown: the points indicating the marginal predicted suitability for *L. italicus* and *L. v. meridionalis* within the sympatric area, the occurrence density curves obtained from the ‘ecospat’ package, and in the upper-right corner a map showing the current values of the respective predictor in the central portion of Apennines including the sympatric area. Most of the occurrence records for both *Lissotriton* species falling within the sympatric area show high predicted suitability associated with intermediate values of the highly contributing predictors (Fig. 3).

We built models for the current scenario, using the previously selected bioclimatic variables (except for BIO5, which was highly correlated with ALT, see Additional file 2) and the topographic ones, in order to assess the importance of the latter. These models resulted in a clear predominance of the climate-related predictors; in fact, ASPECT and SLOPE showed low contribution values for both species. With regard to ALT, even though it resulted as the second highest contributor for *L. italicus* (ALT = 11.2%, 1st predictor: BIO19 = 15.3%), and the third highest contributor for *L. v. meridionalis* (ALT = 9.3%, 1st predictor: BIO17 = 20.9%, 2nd predictor: BIO2 = 10.5%), the corresponding marginal response curves showed a similar and slightly multi-modal trend for both species, probably because of the correlation between this variable and temperature- and precipitation-related ones.

**Fig. 4:**
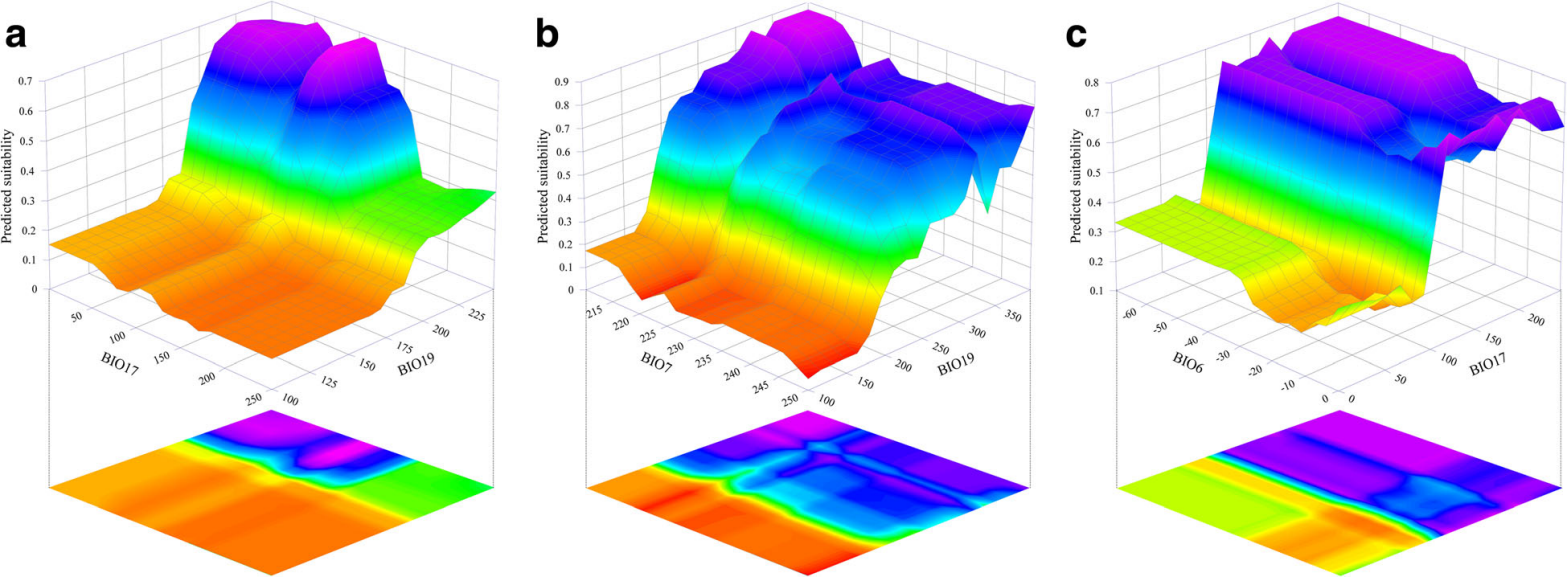
Three-dimensional plots of the most noticeable pairwise interactions. Plotted pairwise interactions, as resulted from the cross-validated BRT models, between **a** BIO17 and BIO19 for *Lissotriton italicus*; **b** BIO7 and BIO19 for *L. italicus*; **c** BIO6 and BIO17 for *L. v. meridionalis*. Areas in blue-violet represent the combined range of values for which predicted suitability is higher

*Lissotriton italicus* model showed relatively high pairwise interaction scores for the pairs BIO19 - BIO17 and BIO7 - BIO19, with relative interaction strength = 62.8 and 31.0, respectively. For *L. v. meridionalis*, the only noticeable, though moderate, pairwise interaction resulted between BIO17 and BIO6 (minimum temperature of the coldest month), with relative interaction strength = 22.5. The corresponding three-dimensional partial dependence plots, showing how the variation of the above-mentioned pairs of predictors influences the modelled suitability, are reported in Fig. 4.

**Fig. 5:**
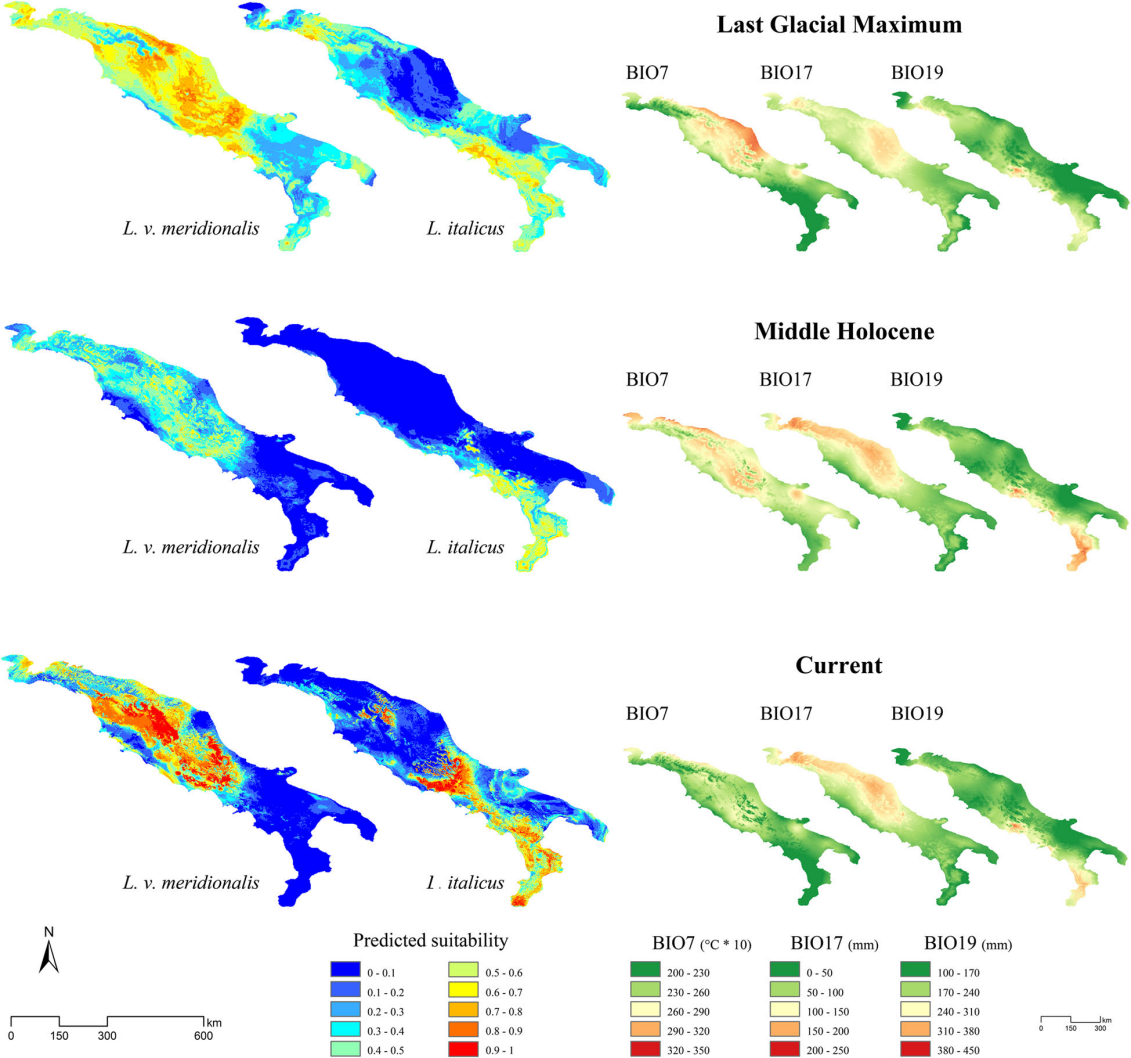
Modelled distributions of both *Lissotriton* species for Last Glacial Maximum, Middle Holocene and current scenarios. Left side: maps of the predicted suitability for *Lissotriton vulgaris meridionalis* and *L. italicus* resulting from the cross-validated BRT models built on the current bioclimatic conditions (last row) and then projected to the Last Glacial Maximum (LGM) and Middle Holocene (MOL) scenarios. Right side: maps showing the values of BIO7, BIO17 and BIO19 within the three temporal scenarios considered; in the maps for the LGM and the MOL scenarios, the values of each variable within the CCSM4 and the MIROC-ESM paleoclimatic reconstructions were averaged.

The averaged maps of predicted suitability for the current, LGM and MOL scenarios based on the set of non-correlated bioclimatic variables, are shown in Fig. 5. The values of BIO7, BIO17 and BIO19 for the three considered scenarios are also reported as separated maps in Fig. 5. These three variables, resulting as shared highly contributing predictors for *L. italicus* and *L. v. meridionalis* from both the BRT and the Maxent models, show noticeable variation among current, LGM and MOL climatic conditions, with an apparent association with the expansion and contraction patterns of predicted suitability modelled for the two species considered.

With respect to the current climatic conditions across the training points, the MESS analysis returned a considerable degree of extrapolation only for the LGM scenarios, particularly for the MIROC-ESM model (Additional file 5). Nonetheless, the presence of non-analog climate within the LGM scenarios does not seem to noticeably affect the projections of the BRT models built on the current conditions when it is accounted for by penalizing the models requiring extrapolation in our weighted average formula, as emerges from the plot in Additional file 5.

**Fig. 6:**
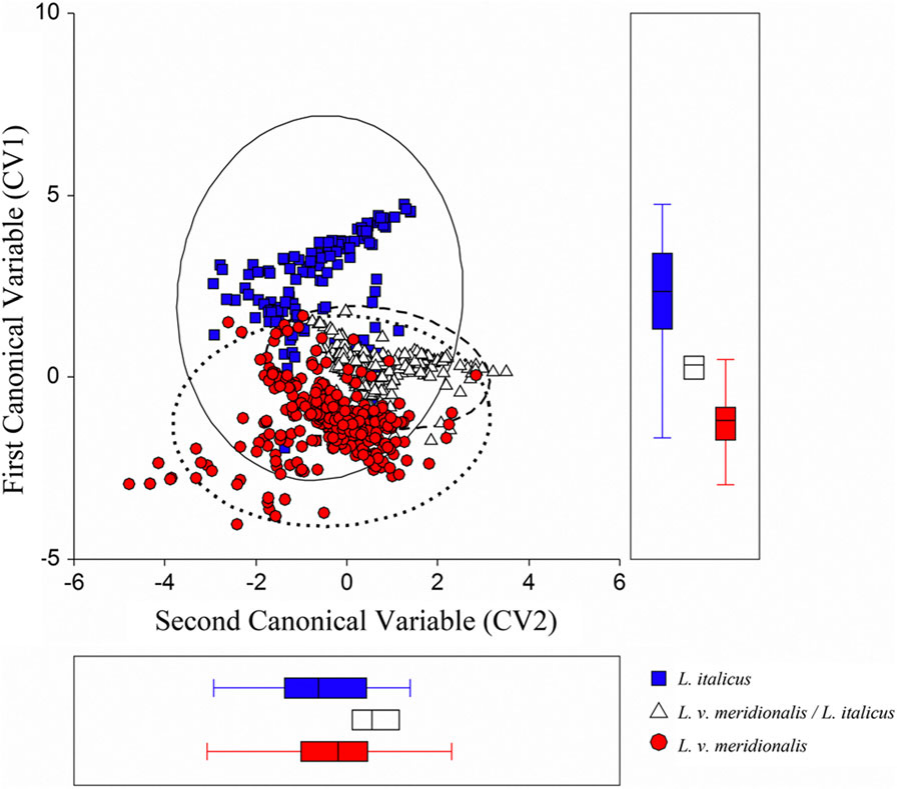
Scatterplot of the two Canonical Variates resulting from the Discriminant Stepwise Analysis. Discriminant Stepwise Analysis: scatterplots (CV1 by CV2) of the Canonical Variates. Analysis performed considering the six most contributing variables from the BRT models for *Lissotriton italicus* and *L. vulgaris meridionalis*. Black contoured triangles correspond to the localities where the two species are found in sympatry (*L. v. meridionalis/L. italicus*).

The forward stepwise discriminant analysis carried out shows that five of the six variables considered are highly discriminating (Table 2 and Fig. 6), while BIO4 (temperature seasonality) is not significant. The relative classification matrix (Table 3) shows a total percentage of 85.5% corrected attributions to the different areas of the two *Lissotriton* species (*L. italicus*: allopatric area = 80.6%, *L. italicus / L. v. meridionalis*: sympatric area = 87.6%, *L. v. meridionalis*: allopatric area = 88.3%).

**Table 2:** Discriminant Stepwise Analysis considering the six most contributing variables within the BRT models obtained

Variables	F to enter	p-level	Lambda
BIO19	191.72	<0.001	30.21
BIO12	133.19	<0.001	23.12
BIO17	66.10	<0.001	12.98
BIO2	55.16	<0.001	10.71
BIO7	13.43	<0.001	2.94

Discriminant Stepwise Analysis for *Lissotriton italicus* and *L. vulgaris meridionalis* in allopatric and sympatric areas, performed considering the five most contributing variables for the two species as resulted from the cross-validated BRT models: “variables in the model”, “F to enter”, p-level and Wilk’s Lambda values

**Table 3:** Classification matrix resulting from the Discriminant Stepwise Analysis

Predicted →	Percent	*L. italicus*	*L. italicus/L. v. meridionalis*	*L. v. meridionalis*	Total
Observed ↓					
*L. italicus*	80.6%	137	15	18	170
*L. italicus/L. v. meridionalis*	87.6%	23	297	19	339
*L. v. meridionalis*	88.3%	13	32	339	384
Total	85.5%	173	344	376	893

Discriminant Stepwise Analysis: classification matrix for *Lissotriton italicus* and *L. vulgaris meridionalis* in allopatric and sympatric areas. Rows: observed classifications; columns: predicted classifications

Both the niche equivalency tests, performed through ENMTools and Ecospat, confirmed significant differences between the environmental niches of the two species. In ENMTools, the observed niche overlap value between the BRT models obtained for the two *Lissotriton* species under the current climatic conditions was *D* = 0.371, falling far outside the 95th percentile of the null distribution (*D*_5%_ = 0.885, *D*_95%_ = 0.914); in Ecospat, instead, the observed niche overlap value was *D* = 0.463 while the null distribution of simulated niche overlaps ranges between *D*_5%_ = 0.591 and *D*_95%_ = 0.700, leading to the rejection of the null hypothesis (*p* < 0.001).

**Fig. 7:**
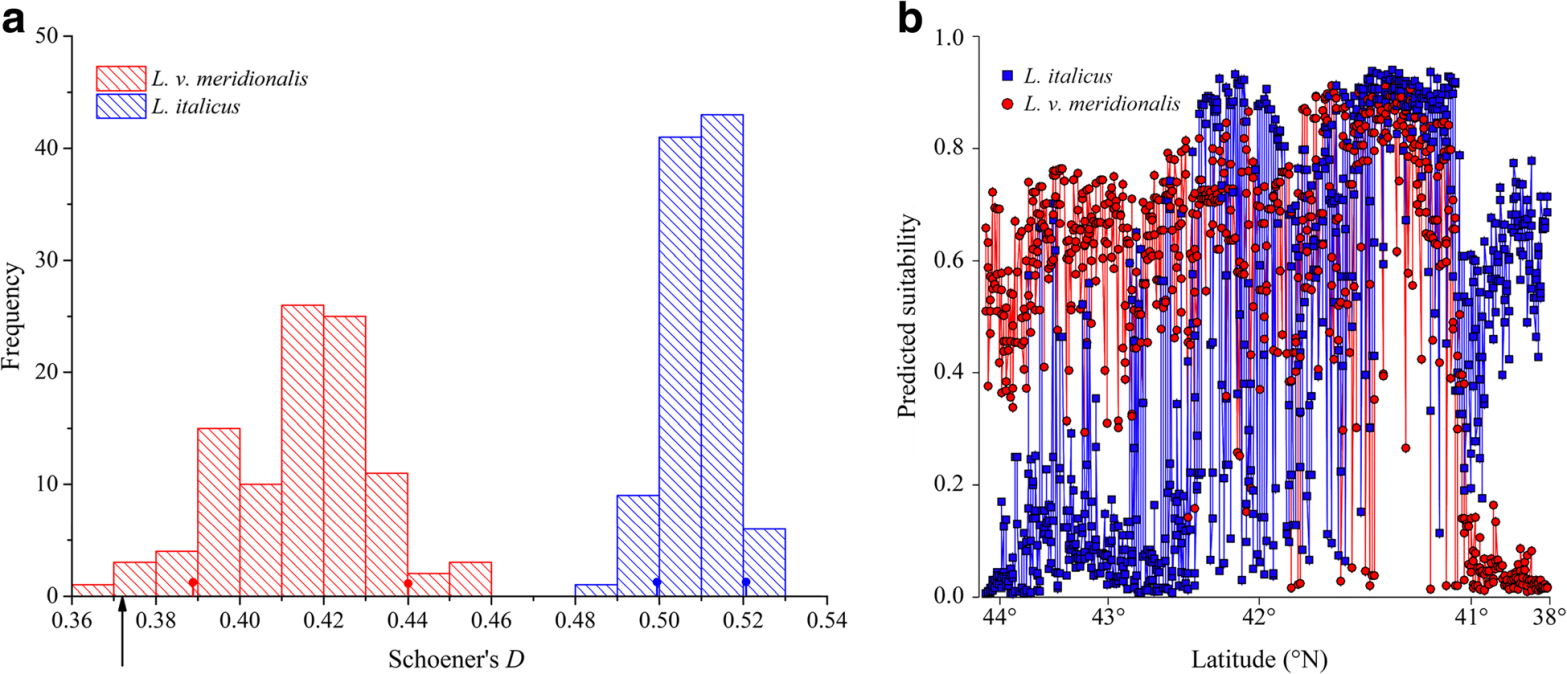
Histograms of Schoener’s *D* and predicted suitability as a function of latitude. **a** Histograms of Schoener’s *D* null distributions obtained from ENMtools background test for *Lissotriton vulgaris meridionalis* (*red*) and *L. italicus* (*blue*), compared with the observed niche overlap value (*black arrow*). Small lines with dots represent the 95th percentile of the null distribution densities for *L. v. meridionalis* (in *red*) and *L. italicus* (in *blue*); **b** predicted suitability as a function of the latitudinal gradient spanning the study area

With respect to the test of niche similarity, the two approaches did not provide fully concordant results: The background test performed in ENMTools resulted in 95th percentile limits of the null distributions *D*_5%_ = 0.386, *D*_95%_ = 0.439 for *L. v. meridionalis* and *D*_5%_ = 0.497, *D*_95%_ = 0.521 for *L. italicus*. Thus, as illustrated in Fig. 7a, the observed niche overlap value (*D* = 0.371) is lower than the 5th percentile of both the null distributions, supporting the hypothesis of niche divergence. Nonetheless, the small gap between the observed overlap value and the 5th percentile of the null distribution resulting from the background test performed for *L. v. meridionalis* suggests that the difference between the environmental niches of *L. v. meridionalis* and *L. italicus* could be mainly due to the climatic conditions characterizing the area of occurrence of this latter. In Ecospat, instead, the null distribution shows 95th percentile limits of *D*_5%_ = 0.062 and *D*_95%_ = 0.512; since the observed overlap value (*D* = 0.463) falls within the 95th percentile of the simulated niche overlaps density, this time the null hypothesis of niche similarity cannot be rejected (*p* = 0.886).

The predicted suitability along the latitudinal gradient spanning the study area, for both *L. v. meridionalis* and *L. italicus*, is shown in Fig. 7b.

## Discussion

Environmental conditions have deeply affected the distributional patterns of many animal groups in southern European peninsulas during the Quaternary (e.g., [70–72]). For instance, climate played an important role in amphibians’ biogeography, as recently observed by Reino et al. [12] for some parapatric anurans.

In agreement with this hypothesis, our comprehensive analysis of the SDMs obtained for *Lissotriton v. meridionalis* and *L. italicus* clearly shows that these species occur preferentially in sites hosting specific bioclimatic conditions, with precipitation-linked variables (i.e. BIO17 and BIO19) and annual temperature variation (BIO7) strongly influencing their past and current distribution. Discriminant analysis and the outputs from the niche equivalency and niche similarity tests furtherly confirm that the environmental niches of these two newts have different peculiarities but are not strongly divergent, consistently with the presence of a sympatric area hosting conditions suitable for both the taxa. The discrepancies between the results obtained from the two niche similarity tests are probably due to the different techniques that the two methods use to select and weight the variables on which niche overlap is then computed [73] (SDM-based for ENMtools and PCA for Ecospat).

The opposite trends shown for the two urodeles by the marginal response curves of the most contributing variables suggest that the SDMs obtained properly distinguished the different sets of bioclimatic conditions favoring or not the presence of each *Lissotriton* species in allopatric or sympatric areas. Precipitation of the driest quarter (BIO17) and precipitation of the coldest quarter (BIO19) are not only the most contributing variables for *L. v. meridionalis* and *L. italicus* respectively, but they also interact each other (see Fig. 3a) and with other bioclimatic variables (see Fig. 3b and c), increasing synergistically the predicted suitability in a certain range. The first and the second interactions shown in Fig. 3a and b reflect some phenological traits of *L. italicus*: this species can mate and lay eggs, depending on microclimatic conditions, during the whole year [24]. With water available even during cold months (i.e. high values of BIO19) and relatively stable annual temperatures (low values of BIO7), *L. italicus* can reproduce during autumn and winter. The low values of precipitation during the driest quarter (i.e. BIO17) associated with relatively high values of predicted suitability (see Fig. 3) are consistent with the reproductive phenology of this species, whose embryo and larval development takes, on average, half of the time with respect to *L. v. meridionalis* [24], so that *L. italicus* individuals generally reach the adult phase before the driest months. Furthermore, *L. italicus* easily aestivate during extremely dry periods [24]. On the contrary, *L. v. meridionalis* is strongly dependent on the precipitation of driest months, because of the longer time span needed for its embryo and larval development, and also because it usually lives in shallow water bodies (up to 50–60 cm of depth), which may rapidly drain during dry periods [23]. The coldest temperatures of the year (BIO6) weakly interact with BIO17 within the BRT model obtained for *L. v. meridionalis* probably because this newt, which can hibernate in water during winter, increasing the probability of a successful reproduction during spring [23], may not survive the harsh winter conditions which occur at higher latitudes with respect to the area inhabited by *L. italicus*. Contrarily to these precipitation-and-temperature-linked variables, topographic predictors were shown to be less informative with respect to the distributional patterns of the two target species.

The potential altitudinal range of the sympatric area located in Central Italy spans from the sea level to almost 3000 m a.s.l., thus including from Mediterranean to alpine habitats. However, the real altitudinal range of the two newts analyzed, based on the available data, goes from 1 to 1650 m a.s.l.. In this area and in this altitudinal interval, highly contributing variables for both the two newts show intermediate values (see Fig. 3), making possible the coexistence of *L. v. meridionalis* and *L. italicus*, coherently with a scenario of niche differentiation driven by the climate.

Particularly interesting is also the evidence of the isolated “patch” with high predicted suitability for *L. italicus* located in the central portion of Northern Apennines (see Fig. 2); it emerges from both the BRT and the Maxent models even though the species has never been detected there. This potentially suitable area may not have been colonized both because of the existence of a climatically unsuitable interposed area and/or because of the presence of *L. v. meridionalis* determining possible competitive displacement.

The projections of the BRT models to the two past scenarios were made under the assumption of temporal niche conservatism [74], since the temporal scale of our hindcasting is relatively short. The MESS analysis confirmed that our projections to past scenarios required some degree of extrapolation only for the LGM. Nonetheless, the transformation of the MES to the MEDI permitted us to verify that the patterns of predicted suitability show minimal changes when model extrapolation is penalized in averaging projections from different GCMs.

Model hindcasting provided some interesting insights into the influence that climatic oscillations during the Late Pleistocene and the Holocene had on the distribution of the two *Lissotriton* species analyzed. The diffused area with medium-to-high values of predicted suitability for *L. v. meridionalis* during the Last Glacial Maximum is linked to the precipitation trends in the Central Apennines. Even if it may seem counter-intuitive, since it is commonly thought that water should not have been fully available in that period due to retention within glaciers, precipitation during the LGM increased in peninsular Italy, because of a regional southerly atmospheric circulation which brought humidity from the central region of the Mediterranean Sea towards the Italian peninsula [75]. These climatic conditions could have allowed *L. v. meridionalis* to move further southwards than Central Apennines, as suggested by some areas in the southern peninsula showing medium suitability values. The past presence of this species at lower latitudes with respect to its actual occurrence area is also confirmed by some fossil records in the Apulian region [76]. On the contrary, the increasing values of the precipitation of the driest quarter within the central and south-western areas of Apennines during the LGM may have determined a shift in the distribution of *L. italicus*, which is predicted by our models to prefer low-to-medium values of precipitation in the driest quarter, towards the drier Adriatic region, which would have been also at lower risk of competition with *L. v. meridionalis* since this latter suffers from dry conditions (see above). This probably has favoured the colonization of the Apulian province, with high values of the temperature annual range hindering a northwards Adriatic colonization. This scenario agrees with what hypothesized by Canestrelli et al. [35] for the differentiation timing of the Apulian *L. italicus* clade.

During the Middle Holocene, the increase of the temperature annual range within the central and south-eastern portion of Apennines determined unfavorable conditions for *L. italicus*, contracting its range to the southern part of the LGM Tyrrhenian corridor, where the medium-to-high values of the precipitation of coldest quarter maintained suitable conditions. The absence of records of this species at latitudes higher than the current sympatric zone supports this scenario. Similarly, the climatic changes during the transition from the Last Glacial Maximum to the interglacial Holocene period seem to have determined the contraction and fragmentation of the potential range for *L. v. meridionalis* within the study area, which is now centered in the central and northern Apennines. The reason of the medium-to-low predicted suitability for both species in the MOL scenario can be found in a general decrease of precipitations in the study area [75, 77, 78], with slight increases only in the north-eastern area of Apennines.

Our results regarding *L. v. meridionalis* distribution during the Last Glacial Maximum also agree with the species’ evolutionary history throughout the Pleistocene inferred by Maura et al. [79]. Their rejection of a scenario of southern refugia with postglacial northwards re-colonization is corroborated by our models. In fact, multiple and separated areas showing medium-to-highly suitable areas can be found in Central and Northern Apennines, supporting the hypothesis of multiple northern glacial refugia.

The comparison of hindcasted SDMs with results from previous studies based on genetic evidence confirmed to be a good strategy to shed light on some of the factors affecting the distributional shifts of the target species over time [32, 80, 81]. Trends of contraction and expansion emerging from our work generally agree with the scenarios of Late Pleistocene glacial refugia well supported in literature [12, 71, 82, 83], especially for the Italian peninsula (e.g., [11, 22, 35, 84]).

## Conclusions

The strong influence of temperature annual range and precipitation-linked variables on the distribution of *Lissotriton v. meridionalis* and *L. italicus* clearly emerges from our SDMs. The effect of climate on the target species’ distribution is further supported by the results emerging from discriminant analysis and niche equivalency and similarity tests. Moreover, the coherence of the SDMs’ hindcasting with respect to the Pleistocene evolutionary scenarios inferred in previous studies for the two species analyzed suggests that the proper implementation of SDMs and the comparison of their outputs with evidences emerging from molecular-based phylogeographical analyses permit to better understand the complex interactions within the different biogeographical processes shaping the distribution of species. Nonetheless, despite the noticeable agreement between molecular phylogeographic reconstructions and patterns of past predicted suitability resulting from the SDMs obtained, it is still possible that some climatic variables individuated as less contributing in the models built for the current scenario may have indeed played a role in shaping the past distributions of the two *Lissotriton* species analyzed.

## Additional files

Additional file 1: Coordinates of the target species presence points. Coordinates of the presences points for *Lissotriton vulgaris meridionalis* and *L. italicus*. The coordinates provided are not at high GPS resolution in order to avoid the risk of illegal withdrawal by poachers who could use published data (DOC 740 kb)

Additional file 2: Correlation matrix. Correlation matrix built to select the non-correlated bioclimatic variables as predictors for the SDMs. Names of the variables with high values of autocorrelation (Pearson’s *r* > 0.85, in red, or *r* < −0.85, in brown) are highlighted in yellow (DOC 91 kb)

Additional file 3: WorldClim bioclimatic variables. Codes and explication, as reported in the WorldClim website, of the nineteen bioclimatic variables considered as candidate predictors (DOC 23 kb)

Additional file 4: Highly contributing variables and predicted suitability maps from the Maxent models. For each *Lissotriton* species are reported the relative contributions of the six most influential predictors and the maps of predicted suitability, as results from the corresponding Maxent models built under the current climatic conditions (DOC 455 kb)

Additional file 5: Extent of model extrapolation and differences in model projections’ averages. The table shows the percent extent of the study area interested by a certain degree of extrapolation (MES < −20) for each combination of species-temporal scenario-GCM; the figure instead illustrates the differences between the raster suitability maps resulting from the weighted and simple average of the SDMs’ projections considering the MIROC-ESM and the CCSM4 paleoclimatic reconstructions (DOC 68 kb)
