# Supplementary material for: Unraveling climate influences on the distribution of the parapatric newts Lissotriton vulgaris meridionalis and L. italicus
Source: Front Zool. 2017 Dec 12;14:55. doi: 10.1186/s12983-017-0239-4 (PMC5727953; doi:10.1186/s12983-017-0239-4)
Supplement: Supplementary file 1 — Coordinates of the target species presence points. Coordinates of the presences points for Lissotriton vulgaris meridionalis and L. italicus. The coordinates provided are not at high GPS resolution in order to avoid the risk of illegal withdrawal by poachers who could use published data (DOC 740 kb) [file 12983_2017_239_MOESM1_ESM.doc]

Additional file 1

| Species name | Longitude | Latitude |
| --- | --- | --- |
| *L. italicus* | 14.5 | 41.5 |
| *L. italicus* | 13.6 | 42.8 |
| *L. italicus* | 14.4 | 41.8 |
| *L. italicus* | 13.6 | 41.3 |
| *L. italicus* | 13.4 | 41.5 |
| *L. italicus* | 13.1 | 41.6 |
| *L. italicus* | 13.9 | 41.2 |
| *L. italicus* | 14.6 | 41.5 |
| *L. italicus* | 14.4 | 41.8 |
| *L. italicus* | 14.1 | 41.9 |
| *L. italicus* | 14.1 | 42.0 |
| *L. italicus* | 14.1 | 42.0 |
| *L. italicus* | 14.1 | 42.0 |
| *L. italicus* | 14.2 | 42.0 |
| *L. italicus* | 14.2 | 42.1 |
| *L. italicus* | 14.2 | 41.9 |
| *L. italicus* | 14.2 | 41.9 |
| *L. italicus* | 14.1 | 41.9 |
| *L. italicus* | 13.9 | 42.2 |
| *L. italicus* | 14.0 | 42.2 |
| *L. italicus* | 13.9 | 42.2 |
| *L. italicus* | 13.9 | 42.2 |
| *L. italicus* | 13.9 | 42.2 |
| *L. italicus* | 14.0 | 42.1 |
| *L. italicus* | 14.0 | 42.2 |
| *L. italicus* | 14.0 | 42.2 |
| *L. italicus* | 14.0 | 42.2 |
| *L. italicus* | 14.0 | 42.2 |
| *L. italicus* | 14.0 | 42.2 |
| *L. italicus* | 14.0 | 42.2 |
| *L. italicus* | 14.0 | 42.2 |
| *L. italicus* | 14.0 | 42.2 |
| *L. italicus* | 14.0 | 42.2 |
| *L. italicus* | 14.0 | 42.2 |
| *L. italicus* | 14.0 | 42.1 |
| *L. italicus* | 14.0 | 42.1 |
| *L. italicus* | 14.1 | 42.1 |
| *L. italicus* | 14.0 | 42.1 |
| *L. italicus* | 14.0 | 42.1 |
| *L. italicus* | 14.0 | 42.1 |
| *L. italicus* | 14.0 | 42.1 |
| *L. italicus* | 14.0 | 42.1 |
| *L. italicus* | 14.0 | 42.1 |
| *L. italicus* | 14.0 | 42.1 |
| *L. italicus* | 14.0 | 42.1 |
| *L. italicus* | 14.0 | 42.1 |
| *L. italicus* | 14.0 | 42.1 |
| *L. italicus* | 14.0 | 42.1 |
| *L. italicus* | 14.0 | 42.1 |
| *L. italicus* | 13.4 | 41.4 |
| *L. italicus* | 14.4 | 41.4 |
| *L. italicus* | 13.6 | 42.7 |
| *L. italicus* | 13.6 | 42.8 |
| *L. italicus* | 13.6 | 42.8 |
| *L. italicus* | 13.6 | 42.8 |
| *L. italicus* | 13.7 | 42.5 |
| *L. italicus* | 13.3 | 41.3 |
| *L. italicus* | 13.7 | 42.5 |
| *L. italicus* | 14.2 | 41.9 |
| *L. italicus* | 14.0 | 42.2 |
| *L. italicus* | 14.1 | 42.0 |
| *L. italicus* | 14.0 | 42.2 |
| *L. italicus* | 13.9 | 42.3 |
| *L. italicus* | 14.0 | 41.7 |
| *L. italicus* | 14.5 | 41.5 |
| *L. italicus* | 13.0 | 41.7 |
| *L. italicus* | 13.0 | 41.7 |
| *L. italicus* | 13.6 | 41.3 |
| *L. italicus* | 13.6 | 41.3 |
| *L. italicus* | 13.7 | 41.3 |
| *L. italicus* | 13.8 | 41.3 |
| *L. italicus* | 13.8 | 41.3 |
| *L. italicus* | 13.7 | 41.3 |
| *L. italicus* | 13.7 | 41.3 |
| *L. italicus* | 13.7 | 41.3 |
| *L. italicus* | 13.7 | 41.3 |
| *L. italicus* | 13.6 | 41.3 |
| *L. italicus* | 13.6 | 41.3 |
| *L. italicus* | 13.7 | 41.3 |
| *L. italicus* | 13.6 | 41.4 |
| *L. italicus* | 13.6 | 41.3 |
| *L. italicus* | 13.6 | 41.3 |
| *L. italicus* | 13.3 | 41.3 |
| *L. italicus* | 13.3 | 41.3 |
| *L. italicus* | 13.2 | 41.3 |
| *L. italicus* | 13.3 | 41.4 |
| *L. italicus* | 13.2 | 41.4 |
| *L. italicus* | 13.3 | 41.4 |
| *L. italicus* | 13.3 | 41.4 |
| *L. italicus* | 13.3 | 41.4 |
| *L. italicus* | 13.3 | 41.4 |
| *L. italicus* | 13.3 | 41.4 |
| *L. italicus* | 13.3 | 41.4 |
| *L. italicus* | 13.4 | 41.4 |
| *L. italicus* | 13.4 | 41.4 |
| *L. italicus* | 13.4 | 41.4 |
| *L. italicus* | 13.4 | 41.4 |
| *L. italicus* | 13.4 | 41.4 |
| *L. italicus* | 13.0 | 41.7 |
| *L. italicus* | 13.1 | 41.7 |
| *L. italicus* | 13.1 | 41.7 |
| *L. italicus* | 13.3 | 41.5 |
| *L. italicus* | 13.4 | 41.5 |
| *L. italicus* | 13.4 | 41.5 |
| *L. italicus* | 13.4 | 41.5 |
| *L. italicus* | 13.4 | 41.5 |
| *L. italicus* | 13.5 | 41.5 |
| *L. italicus* | 13.5 | 41.4 |
| *L. italicus* | 13.7 | 41.4 |
| *L. italicus* | 13.6 | 41.4 |
| *L. italicus* | 13.7 | 41.4 |
| *L. italicus* | 13.8 | 41.4 |
| *L. italicus* | 13.5 | 41.4 |
| *L. italicus* | 13.6 | 41.4 |
| *L. italicus* | 13.5 | 41.4 |
| *L. italicus* | 13.9 | 42.1 |
| *L. italicus* | 14.0 | 42.2 |
| *L. italicus* | 13.7 | 42.8 |
| *L. italicus* | 14.2 | 42.0 |
| *L. italicus* | 13.9 | 42.2 |
| *L. italicus* | 14.2 | 42.0 |
| *L. italicus* | 14.0 | 41.8 |
| *L. italicus* | 14.2 | 42.2 |
| *L. italicus* | 14.1 | 41.8 |
| *L. italicus* | 14.4 | 41.9 |
| *L. italicus* | 14.4 | 41.4 |
| *L. italicus* | 13.6 | 41.3 |
| *L. italicus* | 13.3 | 41.3 |
| *L. italicus* | 13.4 | 41.4 |
| *L. italicus* | 13.5 | 41.4 |
| *L. italicus* | 13.5 | 41.4 |
| *L. italicus* | 13.8 | 41.4 |
| *L. italicus* | 14.0 | 41.6 |
| *L. italicus* | 14.1 | 41.7 |
| *L. italicus* | 14.1 | 41.7 |
| *L. italicus* | 14.6 | 41.5 |
| *L. italicus* | 14.4 | 41.9 |
| *L. italicus* | 14.4 | 41.9 |
| *L. italicus* | 13.2 | 41.3 |
| *L. italicus* | 13.2 | 41.4 |
| *L. italicus* | 13.2 | 41.4 |
| *L. italicus* | 13.2 | 41.4 |
| *L. italicus* | 13.2 | 41.4 |
| *L. italicus* | 13.3 | 41.3 |
| *L. italicus* | 13.3 | 41.4 |
| *L. italicus* | 13.3 | 41.4 |
| *L. italicus* | 13.3 | 41.4 |
| *L. italicus* | 13.3 | 41.4 |
| *L. italicus* | 13.3 | 41.4 |
| *L. italicus* | 13.3 | 41.4 |
| *L. italicus* | 13.3 | 41.4 |
| *L. italicus* | 13.4 | 41.4 |
| *L. italicus* | 13.3 | 41.5 |
| *L. italicus* | 13.4 | 41.4 |
| *L. italicus* | 13.4 | 41.4 |
| *L. italicus* | 13.4 | 41.4 |
| *L. italicus* | 13.4 | 41.4 |
| *L. italicus* | 13.4 | 41.5 |
| *L. italicus* | 13.4 | 41.5 |
| *L. italicus* | 13.5 | 41.5 |
| *L. italicus* | 13.5 | 41.4 |
| *L. italicus* | 13.5 | 41.4 |
| *L. italicus* | 13.4 | 41.4 |
| *L. italicus* | 13.4 | 41.4 |
| *L. italicus* | 13.3 | 41.3 |
| *L. italicus* | 14.2 | 41.9 |
| *L. italicus* | 13.9 | 42.2 |
| *L. italicus* | 14.0 | 42.2 |
| *L. italicus* | 13.9 | 42.2 |
| *L. italicus* | 14.0 | 42.2 |
| *L. italicus* | 14.2 | 42.0 |
| *L. italicus* | 14.2 | 41.9 |
| *L. italicus* | 14.2 | 41.9 |
| *L. italicus* | 14.2 | 41.9 |
| *L. italicus* | 14.1 | 41.9 |
| *L. italicus* | 14.1 | 42.0 |
| *L. italicus* | 14.0 | 42.1 |
| *L. italicus* | 14.0 | 42.1 |
| *L. italicus* | 14.1 | 42.1 |
| *L. italicus* | 14.0 | 42.1 |
| *L. italicus* | 14.0 | 42.1 |
| *L. italicus* | 14.0 | 42.1 |
| *L. italicus* | 14.0 | 42.2 |
| *L. italicus* | 14.0 | 42.2 |
| *L. italicus* | 14.0 | 42.2 |
| *L. italicus* | 14.0 | 42.1 |
| *L. italicus* | 14.2 | 42.0 |
| *L. italicus* | 14.4 | 41.9 |
| *L. italicus* | 14.0 | 42.1 |
| *L. italicus* | 13.7 | 42.8 |
| *L. italicus* | 13.3 | 41.3 |
| *L. italicus* | 13.9 | 42.2 |
| *L. italicus* | 13.8 | 42.4 |
| *L. italicus* | 14.4 | 41.8 |
| *L. italicus* | 14.5 | 41.5 |
| *L. italicus* | 13.4 | 41.4 |
| *L. italicus* | 13.4 | 41.4 |
| *L. italicus* | 13.5 | 41.4 |
| *L. italicus* | 13.6 | 41.3 |
| *L. italicus* | 13.6 | 41.3 |
| *L. italicus* | 13.6 | 41.3 |
| *L. italicus* | 13.6 | 41.3 |
| *L. italicus* | 13.6 | 41.3 |
| *L. italicus* | 13.5 | 41.4 |
| *L. italicus* | 13.6 | 41.4 |
| *L. italicus* | 13.6 | 41.4 |
| *L. italicus* | 13.6 | 41.4 |
| *L. italicus* | 13.7 | 41.3 |
| *L. italicus* | 13.7 | 41.3 |
| *L. italicus* | 13.7 | 41.3 |
| *L. italicus* | 13.7 | 41.4 |
| *L. italicus* | 13.1 | 43.3 |
| *L. italicus* | 13.6 | 42.8 |
| *L. italicus* | 13.6 | 42.7 |
| *L. italicus* | 13.6 | 42.7 |
| *L. italicus* | 13.6 | 42.8 |
| *L. italicus* | 13.8 | 42.6 |
| *L. italicus* | 13.8 | 42.6 |
| *L. italicus* | 13.8 | 42.6 |
| *L. italicus* | 14.2 | 42.0 |
| *L. italicus* | 13.7 | 42.7 |
| *L. italicus* | 14.4 | 42.2 |
| *L. italicus* | 13.9 | 42.5 |
| *L. italicus* | 14.1 | 42.0 |
| *L. italicus* | 13.9 | 42.1 |
| *L. italicus* | 14.1 | 42.0 |
| *L. italicus* | 14.0 | 41.8 |
| *L. italicus* | 14.3 | 42.1 |
| *L. italicus* | 14.1 | 41.7 |
| *L. italicus* | 14.3 | 42.1 |
| *L. italicus* | 14.3 | 42.3 |
| *L. italicus* | 14.4 | 42.1 |
| *L. italicus* | 15.8 | 40.2 |
| *L. italicus* | 15.7 | 40.8 |
| *L. italicus* | 15.8 | 40.5 |
| *L. italicus* | 16.6 | 40.4 |
| *L. italicus* | 16.0 | 40.6 |
| *L. italicus* | 16.3 | 40.4 |
| *L. italicus* | 15.8 | 40.2 |
| *L. italicus* | 15.9 | 38.0 |
| *L. italicus* | 16.0 | 38.0 |
| *L. italicus* | 15.7 | 38.0 |
| *L. italicus* | 16.0 | 38.0 |
| *L. italicus* | 15.9 | 38.0 |
| *L. italicus* | 15.9 | 38.3 |
| *L. italicus* | 16.0 | 38.6 |
| *L. italicus* | 16.1 | 38.0 |
| *L. italicus* | 16.2 | 38.1 |
| *L. italicus* | 16.1 | 38.2 |
| *L. italicus* | 16.2 | 38.3 |
| *L. italicus* | 16.1 | 38.4 |
| *L. italicus* | 15.9 | 39.6 |
| *L. italicus* | 16.0 | 39.6 |
| *L. italicus* | 16.0 | 39.6 |
| *L. italicus* | 16.1 | 39.1 |
| *L. italicus* | 16.1 | 39.2 |
| *L. italicus* | 16.1 | 39.3 |
| *L. italicus* | 16.1 | 39.4 |
| *L. italicus* | 16.1 | 39.5 |
| *L. italicus* | 16.1 | 39.6 |
| *L. italicus* | 16.1 | 39.7 |
| *L. italicus* | 15.8 | 39.9 |
| *L. italicus* | 15.9 | 39.7 |
| *L. italicus* | 15.9 | 39.9 |
| *L. italicus* | 16.0 | 39.9 |
| *L. italicus* | 16.0 | 39.9 |
| *L. italicus* | 16.0 | 39.9 |
| *L. italicus* | 16.0 | 40.1 |
| *L. italicus* | 15.9 | 40.2 |
| *L. italicus* | 16.0 | 40.1 |
| *L. italicus* | 16.0 | 40.1 |
| *L. italicus* | 16.2 | 38.1 |
| *L. italicus* | 16.2 | 38.2 |
| *L. italicus* | 16.2 | 38.6 |
| *L. italicus* | 16.2 | 38.6 |
| *L. italicus* | 16.2 | 38.7 |
| *L. italicus* | 16.4 | 38.3 |
| *L. italicus* | 16.3 | 38.5 |
| *L. italicus* | 16.3 | 38.7 |
| *L. italicus* | 16.3 | 38.7 |
| *L. italicus* | 16.4 | 38.4 |
| *L. italicus* | 16.4 | 38.5 |
| *L. italicus* | 16.4 | 38.8 |
| *L. italicus* | 16.6 | 38.8 |
| *L. italicus* | 16.2 | 39.0 |
| *L. italicus* | 16.3 | 39.0 |
| *L. italicus* | 16.2 | 39.1 |
| *L. italicus* | 16.3 | 39.3 |
| *L. italicus* | 16.2 | 39.4 |
| *L. italicus* | 16.3 | 39.5 |
| *L. italicus* | 16.2 | 39.6 |
| *L. italicus* | 16.1 | 39.7 |
| *L. italicus* | 16.3 | 39.0 |
| *L. italicus* | 16.4 | 39.7 |
| *L. italicus* | 16.5 | 39.3 |
| *L. italicus* | 16.7 | 38.9 |
| *L. italicus* | 16.5 | 39.6 |
| *L. italicus* | 16.8 | 38.9 |
| *L. italicus* | 16.7 | 39.2 |
| *L. italicus* | 16.8 | 39.4 |
| *L. italicus* | 16.8 | 39.4 |
| *L. italicus* | 16.7 | 39.4 |
| *L. italicus* | 16.8 | 39.6 |
| *L. italicus* | 17.0 | 39.2 |
| *L. italicus* | 17.0 | 39.2 |
| *L. italicus* | 17.0 | 39.3 |
| *L. italicus* | 16.9 | 39.4 |
| *L. italicus* | 16.9 | 39.5 |
| *L. italicus* | 16.9 | 39.5 |
| *L. italicus* | 17.1 | 39.1 |
| *L. italicus* | 17.0 | 39.3 |
| *L. italicus* | 17.0 | 39.3 |
| *L. italicus* | 17.0 | 39.5 |
| *L. italicus* | 17.1 | 38.9 |
| *L. italicus* | 17.1 | 39.2 |
| *L. italicus* | 16.2 | 39.8 |
| *L. italicus* | 16.2 | 40.0 |
| *L. italicus* | 16.4 | 39.9 |
| *L. italicus* | 16.4 | 39.9 |
| *L. italicus* | 16.4 | 40.0 |
| *L. italicus* | 16.4 | 40.1 |
| *L. italicus* | 16.5 | 39.8 |
| *L. italicus* | 16.5 | 39.9 |
| *L. italicus* | 16.5 | 39.9 |
| *L. italicus* | 16.5 | 40.0 |
| *L. italicus* | 16.5 | 39.8 |
| *L. italicus* | 16.6 | 40.0 |
| *L. italicus* | 13.9 | 41.1 |
| *L. italicus* | 15.3 | 40.3 |
| *L. italicus* | 13.9 | 41.2 |
| *L. italicus* | 15.6 | 40.1 |
| *L. italicus* | 15.6 | 40.1 |
| *L. italicus* | 15.1 | 40.4 |
| *L. italicus* | 15.1 | 40.2 |
| *L. italicus* | 15.3 | 40.3 |
| *L. italicus* | 15.3 | 40.3 |
| *L. italicus* | 14.8 | 41.0 |
| *L. italicus* | 14.8 | 41.0 |
| *L. italicus* | 15.1 | 40.5 |
| *L. italicus* | 15.3 | 40.5 |
| *L. italicus* | 15.4 | 40.4 |
| *L. italicus* | 15.4 | 40.4 |
| *L. italicus* | 15.1 | 40.8 |
| *L. italicus* | 14.5 | 40.9 |
| *L. italicus* | 13.9 | 41.2 |
| *L. italicus* | 15.0 | 40.2 |
| *L. italicus* | 14.8 | 41.0 |
| *L. italicus* | 13.9 | 41.1 |
| *L. italicus* | 15.3 | 40.3 |
| *L. italicus* | 15.3 | 40.3 |
| *L. italicus* | 15.3 | 40.5 |
| *L. italicus* | 14.5 | 40.7 |
| *L. italicus* | 15.3 | 40.5 |
| *L. italicus* | 15.3 | 40.6 |
| *L. italicus* | 15.4 | 40.1 |
| *L. italicus* | 14.8 | 40.9 |
| *L. italicus* | 14.8 | 41.0 |
| *L. italicus* | 15.1 | 40.8 |
| *L. italicus* | 15.2 | 40.8 |
| *L. italicus* | 14.3 | 41.4 |
| *L. italicus* | 14.8 | 40.9 |
| *L. italicus* | 15.1 | 40.8 |
| *L. italicus* | 15.0 | 40.7 |
| *L. italicus* | 14.2 | 41.1 |
| *L. italicus* | 13.7 | 41.3 |
| *L. italicus* | 13.3 | 41.3 |
| *L. italicus* | 13.3 | 41.4 |
| *L. italicus* | 13.3 | 41.4 |
| *L. italicus* | 13.5 | 41.4 |
| *L. italicus* | 13.7 | 41.4 |
| *L. italicus* | 14.0 | 41.5 |
| *L. italicus* | 18.2 | 40.4 |
| *L. italicus* | 17.1 | 40.8 |
| *L. italicus* | 15.7 | 41.3 |
| *L. italicus* | 15.9 | 41.3 |
| *L. italicus* | 15.9 | 41.3 |
| *L. italicus* | 15.2 | 41.8 |
| *L. italicus* | 15.6 | 41.8 |
| *L. italicus* | 15.0 | 41.5 |
| *L. italicus* | 17.6 | 40.6 |
| *L. italicus* | 17.7 | 40.5 |
| *L. italicus* | 16.9 | 41.0 |
| *L. italicus* | 18.1 | 40.5 |
| *L. italicus* | 18.1 | 40.5 |
| *L. italicus* | 17.4 | 40.6 |
| *L. italicus* | 16.1 | 41.3 |
| *L. italicus* | 15.0 | 41.4 |
| *L. italicus* | 15.0 | 41.4 |
| *L. italicus* | 15.1 | 41.4 |
| *L. italicus* | 15.4 | 41.4 |
| *L. italicus* | 15.3 | 41.9 |
| *L. italicus* | 17.8 | 40.7 |
| *L. italicus* | 15.0 | 41.5 |
| *L. italicus* | 15.2 | 41.5 |
| *L. italicus* | 16.0 | 41.9 |
| *L. italicus* | 17.7 | 40.7 |
| *L. italicus* | 13.0 | 43.4 |
| *L. italicus* | 14.1 | 41.7 |
| *L. italicus* | 14.1 | 41.7 |
| *L. italicus* | 14.6 | 41.5 |
| *L. italicus* | 14.6 | 41.5 |
| *L. v. meridionalis* | 13.9 | 41.8 |
| *L. v. meridionalis* | 12.8 | 42.3 |
| *L. v. meridionalis* | 12.7 | 42.4 |
| *L. v. meridionalis* | 14.5 | 41.5 |
| *L. v. meridionalis* | 13.0 | 43.0 |
| *L. v. meridionalis* | 14.6 | 41.5 |
| *L. v. meridionalis* | 14.1 | 42.0 |
| *L. v. meridionalis* | 14.1 | 41.9 |
| *L. v. meridionalis* | 14.2 | 41.9 |
| *L. v. meridionalis* | 13.0 | 42.2 |
| *L. v. meridionalis* | 13.0 | 42.2 |
| *L. v. meridionalis* | 12.8 | 42.1 |
| *L. v. meridionalis* | 12.8 | 42.1 |
| *L. v. meridionalis* | 12.9 | 42.1 |
| *L. v. meridionalis* | 12.8 | 42.1 |
| *L. v. meridionalis* | 12.9 | 42.1 |
| *L. v. meridionalis* | 13.5 | 41.4 |
| *L. v. meridionalis* | 13.5 | 41.4 |
| *L. v. meridionalis* | 13.6 | 42.0 |
| *L. v. meridionalis* | 14.4 | 41.4 |
| *L. v. meridionalis* | 14.3 | 41.9 |
| *L. v. meridionalis* | 14.1 | 41.7 |
| *L. v. meridionalis* | 13.8 | 42.4 |
| *L. v. meridionalis* | 13.6 | 42.0 |
| *L. v. meridionalis* | 13.4 | 42.0 |
| *L. v. meridionalis* | 13.0 | 42.3 |
| *L. v. meridionalis* | 13.0 | 42.3 |
| *L. v. meridionalis* | 13.1 | 42.1 |
| *L. v. meridionalis* | 13.1 | 42.1 |
| *L. v. meridionalis* | 13.4 | 42.2 |
| *L. v. meridionalis* | 14.1 | 42.0 |
| *L. v. meridionalis* | 14.2 | 41.9 |
| *L. v. meridionalis* | 14.1 | 42.0 |
| *L. v. meridionalis* | 14.5 | 41.5 |
| *L. v. meridionalis* | 13.5 | 41.3 |
| *L. v. meridionalis* | 13.6 | 41.3 |
| *L. v. meridionalis* | 13.8 | 41.3 |
| *L. v. meridionalis* | 13.6 | 41.4 |
| *L. v. meridionalis* | 13.5 | 41.4 |
| *L. v. meridionalis* | 13.5 | 41.4 |
| *L. v. meridionalis* | 13.5 | 41.4 |
| *L. v. meridionalis* | 12.9 | 41.7 |
| *L. v. meridionalis* | 13.0 | 41.7 |
| *L. v. meridionalis* | 13.5 | 41.4 |
| *L. v. meridionalis* | 13.5 | 41.4 |
| *L. v. meridionalis* | 13.5 | 41.4 |
| *L. v. meridionalis* | 13.5 | 41.4 |
| *L. v. meridionalis* | 13.5 | 41.4 |
| *L. v. meridionalis* | 13.5 | 41.4 |
| *L. v. meridionalis* | 13.5 | 41.4 |
| *L. v. meridionalis* | 13.6 | 41.4 |
| *L. v. meridionalis* | 13.2 | 41.5 |
| *L. v. meridionalis* | 13.3 | 41.5 |
| *L. v. meridionalis* | 13.4 | 41.5 |
| *L. v. meridionalis* | 13.4 | 41.5 |
| *L. v. meridionalis* | 13.4 | 41.5 |
| *L. v. meridionalis* | 13.4 | 41.5 |
| *L. v. meridionalis* | 13.4 | 41.5 |
| *L. v. meridionalis* | 13.5 | 41.5 |
| *L. v. meridionalis* | 13.5 | 41.5 |
| *L. v. meridionalis* | 13.0 | 41.6 |
| *L. v. meridionalis* | 13.0 | 41.6 |
| *L. v. meridionalis* | 13.0 | 41.7 |
| *L. v. meridionalis* | 13.0 | 41.7 |
| *L. v. meridionalis* | 14.0 | 41.8 |
| *L. v. meridionalis* | 14.1 | 41.8 |
| *L. v. meridionalis* | 14.1 | 41.8 |
| *L. v. meridionalis* | 13.4 | 42.1 |
| *L. v. meridionalis* | 14.1 | 41.8 |
| *L. v. meridionalis* | 14.2 | 42.0 |
| *L. v. meridionalis* | 13.4 | 42.1 |
| *L. v. meridionalis* | 11.6 | 42.4 |
| *L. v. meridionalis* | 12.5 | 41.9 |
| *L. v. meridionalis* | 12.7 | 41.8 |
| *L. v. meridionalis* | 12.9 | 42.1 |
| *L. v. meridionalis* | 12.5 | 41.9 |
| *L. v. meridionalis* | 12.3 | 41.8 |
| *L. v. meridionalis* | 12.8 | 41.5 |
| *L. v. meridionalis* | 13.3 | 41.4 |
| *L. v. meridionalis* | 13.3 | 41.4 |
| *L. v. meridionalis* | 12.7 | 41.6 |
| *L. v. meridionalis* | 12.6 | 42.3 |
| *L. v. meridionalis* | 13.0 | 41.6 |
| *L. v. meridionalis* | 13.3 | 42.7 |
| *L. v. meridionalis* | 13.3 | 42.7 |
| *L. v. meridionalis* | 13.3 | 42.8 |
| *L. v. meridionalis* | 12.2 | 42.4 |
| *L. v. meridionalis* | 13.6 | 41.3 |
| *L. v. meridionalis* | 13.1 | 42.0 |
| *L. v. meridionalis* | 12.1 | 42.0 |
| *L. v. meridionalis* | 13.1 | 42.2 |
| *L. v. meridionalis* | 13.0 | 42.3 |
| *L. v. meridionalis* | 12.2 | 42.4 |
| *L. v. meridionalis* | 13.6 | 41.4 |
| *L. v. meridionalis* | 11.9 | 42.8 |
| *L. v. meridionalis* | 12.4 | 42.2 |
| *L. v. meridionalis* | 11.9 | 42.6 |
| *L. v. meridionalis* | 12.8 | 42.4 |
| *L. v. meridionalis* | 12.7 | 42.4 |
| *L. v. meridionalis* | 12.8 | 42.3 |
| *L. v. meridionalis* | 12.8 | 42.6 |
| *L. v. meridionalis* | 12.1 | 42.6 |
| *L. v. meridionalis* | 13.1 | 41.6 |
| *L. v. meridionalis* | 13.0 | 41.7 |
| *L. v. meridionalis* | 11.8 | 42.6 |
| *L. v. meridionalis* | 12.9 | 42.4 |
| *L. v. meridionalis* | 13.1 | 41.6 |
| *L. v. meridionalis* | 13.1 | 42.2 |
| *L. v. meridionalis* | 12.6 | 43.0 |
| *L. v. meridionalis* | 12.3 | 43.3 |
| *L. v. meridionalis* | 12.3 | 43.5 |
| *L. v. meridionalis* | 12.6 | 43.4 |
| *L. v. meridionalis* | 12.6 | 43.1 |
| *L. v. meridionalis* | 12.8 | 43.1 |
| *L. v. meridionalis* | 12.6 | 42.9 |
| *L. v. meridionalis* | 12.8 | 42.9 |
| *L. v. meridionalis* | 12.9 | 42.8 |
| *L. v. meridionalis* | 12.6 | 42.4 |
| *L. v. meridionalis* | 12.8 | 42.9 |
| *L. v. meridionalis* | 12.8 | 43.3 |
| *L. v. meridionalis* | 12.1 | 43.0 |
| *L. v. meridionalis* | 12.6 | 43.2 |
| *L. v. meridionalis* | 12.4 | 43.1 |
| *L. v. meridionalis* | 12.2 | 43.2 |
| *L. v. meridionalis* | 12.0 | 43.0 |
| *L. v. meridionalis* | 12.2 | 43.3 |
| *L. v. meridionalis* | 12.3 | 42.9 |
| *L. v. meridionalis* | 12.4 | 42.9 |
| *L. v. meridionalis* | 12.6 | 43.2 |
| *L. v. meridionalis* | 12.4 | 43.1 |
| *L. v. meridionalis* | 12.9 | 42.9 |
| *L. v. meridionalis* | 12.6 | 43.0 |
| *L. v. meridionalis* | 12.7 | 43.0 |
| *L. v. meridionalis* | 13.1 | 42.8 |
| *L. v. meridionalis* | 12.2 | 43.2 |
| *L. v. meridionalis* | 12.1 | 43.2 |
| *L. v. meridionalis* | 12.3 | 43.5 |
| *L. v. meridionalis* | 13.1 | 42.8 |
| *L. v. meridionalis* | 12.7 | 42.6 |
| *L. v. meridionalis* | 12.4 | 43.1 |
| *L. v. meridionalis* | 12.0 | 42.8 |
| *L. v. meridionalis* | 12.4 | 43.1 |
| *L. v. meridionalis* | 12.8 | 43.3 |
| *L. v. meridionalis* | 12.7 | 43.4 |
| *L. v. meridionalis* | 13.1 | 42.8 |
| *L. v. meridionalis* | 12.6 | 43.4 |
| *L. v. meridionalis* | 12.3 | 43.5 |
| *L. v. meridionalis* | 12.4 | 43.1 |
| *L. v. meridionalis* | 12.3 | 43.5 |
| *L. v. meridionalis* | 13.0 | 43.5 |
| *L. v. meridionalis* | 12.9 | 43.4 |
| *L. v. meridionalis* | 14.6 | 41.5 |
| *L. v. meridionalis* | 11.1 | 42.7 |
| *L. v. meridionalis* | 11.1 | 42.7 |
| *L. v. meridionalis* | 11.5 | 42.8 |
| *L. v. meridionalis* | 11.7 | 42.8 |
| *L. v. meridionalis* | 11.6 | 42.9 |
| *L. v. meridionalis* | 11.8 | 43.0 |
| *L. v. meridionalis* | 11.9 | 43.0 |
| *L. v. meridionalis* | 12.0 | 43.7 |
| *L. v. meridionalis* | 12.1 | 43.7 |
| *L. v. meridionalis* | 12.1 | 43.7 |
| *L. v. meridionalis* | 13.2 | 41.3 |
| *L. v. meridionalis* | 13.4 | 41.4 |
| *L. v. meridionalis* | 13.4 | 41.4 |
| *L. v. meridionalis* | 13.4 | 41.4 |
| *L. v. meridionalis* | 13.4 | 41.5 |
| *L. v. meridionalis* | 13.4 | 41.5 |
| *L. v. meridionalis* | 13.5 | 41.5 |
| *L. v. meridionalis* | 13.5 | 41.4 |
| *L. v. meridionalis* | 13.5 | 41.4 |
| *L. v. meridionalis* | 13.4 | 41.4 |
| *L. v. meridionalis* | 13.5 | 41.5 |
| *L. v. meridionalis* | 11.2 | 43.1 |
| *L. v. meridionalis* | 14.1 | 42.0 |
| *L. v. meridionalis* | 14.0 | 42.0 |
| *L. v. meridionalis* | 13.1 | 42.0 |
| *L. v. meridionalis* | 13.1 | 41.9 |
| *L. v. meridionalis* | 13.2 | 41.9 |
| *L. v. meridionalis* | 13.3 | 42.0 |
| *L. v. meridionalis* | 13.2 | 41.9 |
| *L. v. meridionalis* | 14.4 | 41.9 |
| *L. v. meridionalis* | 13.5 | 41.4 |
| *L. v. meridionalis* | 13.5 | 41.4 |
| *L. v. meridionalis* | 14.0 | 41.7 |
| *L. v. meridionalis* | 13.4 | 41.4 |
| *L. v. meridionalis* | 13.5 | 41.4 |
| *L. v. meridionalis* | 13.5 | 41.4 |
| *L. v. meridionalis* | 13.5 | 41.4 |
| *L. v. meridionalis* | 13.5 | 41.4 |
| *L. v. meridionalis* | 13.5 | 41.4 |
| *L. v. meridionalis* | 13.5 | 41.4 |
| *L. v. meridionalis* | 13.5 | 41.4 |
| *L. v. meridionalis* | 13.5 | 41.4 |
| *L. v. meridionalis* | 13.5 | 41.3 |
| *L. v. meridionalis* | 13.6 | 41.3 |
| *L. v. meridionalis* | 13.6 | 41.3 |
| *L. v. meridionalis* | 13.5 | 41.4 |
| *L. v. meridionalis* | 13.1 | 42.3 |
| *L. v. meridionalis* | 12.5 | 43.2 |
| *L. v. meridionalis* | 14.1 | 41.8 |
| *L. v. meridionalis* | 14.1 | 41.7 |
| *L. v. meridionalis* | 13.6 | 42.0 |
| *L. v. meridionalis* | 13.0 | 43.0 |
| *L. v. meridionalis* | 13.0 | 43.1 |
| *L. v. meridionalis* | 13.2 | 42.8 |
| *L. v. meridionalis* | 13.3 | 42.7 |
| *L. v. meridionalis* | 13.3 | 42.7 |
| *L. v. meridionalis* | 12.8 | 41.8 |
| *L. v. meridionalis* | 12.1 | 43.7 |
| *L. v. meridionalis* | 12.1 | 43.7 |
| *L. v. meridionalis* | 12.3 | 43.8 |
| *L. v. meridionalis* | 12.5 | 43.7 |
| *L. v. meridionalis* | 13.6 | 42.4 |
| *L. v. meridionalis* | 13.6 | 42.4 |
| *L. v. meridionalis* | 13.8 | 42.0 |
| *L. v. meridionalis* | 13.8 | 42.0 |
| *L. v. meridionalis* | 13.8 | 42.0 |
| *L. v. meridionalis* | 12.0 | 43.7 |
| *L. v. meridionalis* | 12.0 | 43.7 |
| *L. v. meridionalis* | 12.1 | 43.7 |
| *L. v. meridionalis* | 13.6 | 42.2 |
| *L. v. meridionalis* | 13.6 | 42.2 |
| *L. v. meridionalis* | 13.6 | 42.2 |
| *L. v. meridionalis* | 13.7 | 42.1 |
| *L. v. meridionalis* | 13.6 | 42.2 |
| *L. v. meridionalis* | 13.6 | 42.2 |
| *L. v. meridionalis* | 13.3 | 42.4 |
| *L. v. meridionalis* | 13.6 | 42.4 |
| *L. v. meridionalis* | 14.0 | 41.8 |
| *L. v. meridionalis* | 13.9 | 42.1 |
| *L. v. meridionalis* | 14.1 | 41.7 |
| *L. v. meridionalis* | 14.1 | 41.7 |
| *L. v. meridionalis* | 13.4 | 42.1 |
| *L. v. meridionalis* | 14.1 | 41.8 |
| *L. v. meridionalis* | 14.1 | 42.0 |
| *L. v. meridionalis* | 13.4 | 42.1 |
| *L. v. meridionalis* | 13.9 | 41.1 |
| *L. v. meridionalis* | 14.0 | 41.2 |
| *L. v. meridionalis* | 14.8 | 40.9 |
| *L. v. meridionalis* | 14.0 | 41.0 |
| *L. v. meridionalis* | 14.3 | 40.8 |
| *L. v. meridionalis* | 9.6 | 44.7 |
| *L. v. meridionalis* | 12.4 | 43.9 |
| *L. v. meridionalis* | 12.0 | 44.1 |
| *L. v. meridionalis* | 12.7 | 43.9 |
| *L. v. meridionalis* | 12.7 | 43.9 |
| *L. v. meridionalis* | 11.1 | 44.1 |
| *L. v. meridionalis* | 11.3 | 44.2 |
| *L. v. meridionalis* | 12.1 | 44.0 |
| *L. v. meridionalis* | 10.2 | 44.5 |
| *L. v. meridionalis* | 10.6 | 44.4 |
| *L. v. meridionalis* | 10.9 | 44.2 |
| *L. v. meridionalis* | 10.3 | 44.4 |
| *L. v. meridionalis* | 9.8 | 44.5 |
| *L. v. meridionalis* | 9.8 | 44.6 |
| *L. v. meridionalis* | 12.0 | 43.9 |
| *L. v. meridionalis* | 12.3 | 43.9 |
| *L. v. meridionalis* | 12.1 | 44.0 |
| *L. v. meridionalis* | 12.4 | 44.0 |
| *L. v. meridionalis* | 12.0 | 44.1 |
| *L. v. meridionalis* | 12.1 | 43.8 |
| *L. v. meridionalis* | 12.3 | 44.0 |
| *L. v. meridionalis* | 10.3 | 44.4 |
| *L. v. meridionalis* | 9.6 | 44.6 |
| *L. v. meridionalis* | 9.5 | 44.9 |
| *L. v. meridionalis* | 9.8 | 44.9 |
| *L. v. meridionalis* | 9.6 | 44.6 |
| *L. v. meridionalis* | 9.5 | 44.8 |
| *L. v. meridionalis* | 9.6 | 44.6 |
| *L. v. meridionalis* | 9.4 | 45.0 |
| *L. v. meridionalis* | 9.5 | 44.9 |
| *L. v. meridionalis* | 11.1 | 44.3 |
| *L. v. meridionalis* | 11.5 | 44.1 |
| *L. v. meridionalis* | 11.0 | 44.5 |
| *L. v. meridionalis* | 9.7 | 44.5 |
| *L. v. meridionalis* | 9.8 | 44.5 |
| *L. v. meridionalis* | 11.3 | 44.3 |
| *L. v. meridionalis* | 11.3 | 44.3 |
| *L. v. meridionalis* | 11.3 | 44.3 |
| *L. v. meridionalis* | 11.5 | 44.4 |
| *L. v. meridionalis* | 11.7 | 43.9 |
| *L. v. meridionalis* | 12.0 | 43.9 |
| *L. v. meridionalis* | 11.9 | 43.9 |
| *L. v. meridionalis* | 11.9 | 44.2 |
| *L. v. meridionalis* | 11.9 | 43.8 |
| *L. v. meridionalis* | 12.1 | 44.1 |
| *L. v. meridionalis* | 11.6 | 42.3 |
| *L. v. meridionalis* | 12.5 | 41.9 |
| *L. v. meridionalis* | 12.5 | 41.9 |
| *L. v. meridionalis* | 13.3 | 41.7 |
| *L. v. meridionalis* | 12.3 | 41.7 |
| *L. v. meridionalis* | 13.1 | 41.3 |
| *L. v. meridionalis* | 12.3 | 42.2 |
| *L. v. meridionalis* | 12.2 | 42.1 |
| *L. v. meridionalis* | 12.2 | 42.2 |
| *L. v. meridionalis* | 12.7 | 41.8 |
| *L. v. meridionalis* | 12.9 | 42.1 |
| *L. v. meridionalis* | 12.6 | 42.0 |
| *L. v. meridionalis* | 12.5 | 41.9 |
| *L. v. meridionalis* | 12.8 | 41.4 |
| *L. v. meridionalis* | 12.8 | 42.0 |
| *L. v. meridionalis* | 12.6 | 42.2 |
| *L. v. meridionalis* | 12.7 | 42.1 |
| *L. v. meridionalis* | 13.0 | 41.6 |
| *L. v. meridionalis* | 13.1 | 42.0 |
| *L. v. meridionalis* | 13.3 | 41.7 |
| *L. v. meridionalis* | 12.8 | 42.5 |
| *L. v. meridionalis* | 12.8 | 42.5 |
| *L. v. meridionalis* | 12.8 | 42.5 |
| *L. v. meridionalis* | 13.3 | 42.6 |
| *L. v. meridionalis* | 12.5 | 41.9 |
| *L. v. meridionalis* | 11.9 | 42.2 |
| *L. v. meridionalis* | 12.0 | 42.1 |
| *L. v. meridionalis* | 12.1 | 42.3 |
| *L. v. meridionalis* | 12.1 | 41.9 |
| *L. v. meridionalis* | 12.9 | 42.4 |
| *L. v. meridionalis* | 13.2 | 42.7 |
| *L. v. meridionalis* | 13.2 | 42.7 |
| *L. v. meridionalis* | 12.1 | 42.3 |
| *L. v. meridionalis* | 13.7 | 41.3 |
| *L. v. meridionalis* | 13.0 | 42.1 |
| *L. v. meridionalis* | 13.0 | 42.0 |
| *L. v. meridionalis* | 12.1 | 41.9 |
| *L. v. meridionalis* | 13.1 | 42.1 |
| *L. v. meridionalis* | 13.0 | 42.2 |
| *L. v. meridionalis* | 12.1 | 42.3 |
| *L. v. meridionalis* | 13.3 | 41.4 |
| *L. v. meridionalis* | 13.5 | 41.4 |
| *L. v. meridionalis* | 11.9 | 42.7 |
| *L. v. meridionalis* | 12.1 | 42.3 |
| *L. v. meridionalis* | 11.9 | 42.7 |
| *L. v. meridionalis* | 12.4 | 42.1 |
| *L. v. meridionalis* | 12.8 | 41.5 |
| *L. v. meridionalis* | 12.7 | 42.3 |
| *L. v. meridionalis* | 12.8 | 42.3 |
| *L. v. meridionalis* | 12.8 | 42.6 |
| *L. v. meridionalis* | 12.3 | 42.1 |
| *L. v. meridionalis* | 12.5 | 41.6 |
| *L. v. meridionalis* | 11.9 | 42.2 |
| *L. v. meridionalis* | 11.9 | 42.6 |
| *L. v. meridionalis* | 12.0 | 42.7 |
| *L. v. meridionalis* | 12.2 | 42.1 |
| *L. v. meridionalis* | 12.0 | 42.5 |
| *L. v. meridionalis* | 13.2 | 41.5 |
| *L. v. meridionalis* | 13.2 | 41.5 |
| *L. v. meridionalis* | 13.2 | 41.5 |
| *L. v. meridionalis* | 13.0 | 41.5 |
| *L. v. meridionalis* | 13.0 | 41.7 |
| *L. v. meridionalis* | 13.1 | 41.7 |
| *L. v. meridionalis* | 13.1 | 41.9 |
| *L. v. meridionalis* | 12.1 | 42.3 |
| *L. v. meridionalis* | 12.3 | 42.1 |
| *L. v. meridionalis* | 12.2 | 42.4 |
| *L. v. meridionalis* | 11.7 | 42.4 |
| *L. v. meridionalis* | 11.9 | 42.6 |
| *L. v. meridionalis* | 12.5 | 42.3 |
| *L. v. meridionalis* | 12.1 | 42.7 |
| *L. v. meridionalis* | 12.9 | 42.4 |
| *L. v. meridionalis* | 13.2 | 41.6 |
| *L. v. meridionalis* | 13.2 | 41.7 |
| *L. v. meridionalis* | 13.0 | 42.1 |
| *L. v. meridionalis* | 8.3 | 44.4 |
| *L. v. meridionalis* | 8.5 | 44.4 |
| *L. v. meridionalis* | 10.0 | 44.1 |
| *L. v. meridionalis* | 10.0 | 44.1 |
| *L. v. meridionalis* | 10.0 | 44.1 |
| *L. v. meridionalis* | 9.4 | 44.5 |
| *L. v. meridionalis* | 9.2 | 44.9 |
| *L. v. meridionalis* | 9.2 | 44.7 |
| *L. v. meridionalis* | 9.3 | 44.8 |
| *L. v. meridionalis* | 9.2 | 44.7 |
| *L. v. meridionalis* | 9.3 | 44.8 |
| *L. v. meridionalis* | 8.3 | 44.9 |
| *L. v. meridionalis* | 8.3 | 44.8 |
| *L. v. meridionalis* | 9.1 | 44.8 |
| *L. v. meridionalis* | 8.8 | 44.7 |
| *L. v. meridionalis* | 8.2 | 44.9 |
| *L. v. meridionalis* | 8.3 | 44.8 |
| *L. v. meridionalis* | 8.4 | 44.6 |
| *L. v. meridionalis* | 12.6 | 43.0 |
| *L. v. meridionalis* | 12.4 | 43.3 |
| *L. v. meridionalis* | 12.3 | 43.5 |
| *L. v. meridionalis* | 12.6 | 43.3 |
| *L. v. meridionalis* | 12.6 | 43.1 |
| *L. v. meridionalis* | 12.6 | 42.9 |
| *L. v. meridionalis* | 12.7 | 42.8 |
| *L. v. meridionalis* | 12.8 | 42.7 |
| *L. v. meridionalis* | 12.6 | 42.4 |
| *L. v. meridionalis* | 12.7 | 43.4 |
| *L. v. meridionalis* | 12.2 | 43.0 |
| *L. v. meridionalis* | 12.6 | 43.2 |
| *L. v. meridionalis* | 12.5 | 43.1 |
| *L. v. meridionalis* | 12.1 | 43.3 |
| *L. v. meridionalis* | 12.0 | 43.0 |
| *L. v. meridionalis* | 12.1 | 43.3 |
| *L. v. meridionalis* | 12.4 | 42.9 |
| *L. v. meridionalis* | 12.4 | 42.9 |
| *L. v. meridionalis* | 12.6 | 43.2 |
| *L. v. meridionalis* | 12.5 | 43.1 |
| *L. v. meridionalis* | 12.6 | 43.0 |
| *L. v. meridionalis* | 12.7 | 42.9 |
| *L. v. meridionalis* | 13.2 | 42.8 |
| *L. v. meridionalis* | 12.1 | 43.2 |
| *L. v. meridionalis* | 11.9 | 43.1 |
| *L. v. meridionalis* | 12.3 | 43.5 |
| *L. v. meridionalis* | 13.2 | 42.8 |
| *L. v. meridionalis* | 12.8 | 42.6 |
| *L. v. meridionalis* | 12.5 | 43.1 |
| *L. v. meridionalis* | 12.0 | 42.8 |
| *L. v. meridionalis* | 12.5 | 43.1 |
| *L. v. meridionalis* | 12.8 | 43.2 |
| *L. v. meridionalis* | 12.7 | 43.4 |
| *L. v. meridionalis* | 13.2 | 42.8 |
| *L. v. meridionalis* | 12.6 | 43.3 |
| *L. v. meridionalis* | 12.3 | 43.5 |
| *L. v. meridionalis* | 12.5 | 43.1 |
| *L. v. meridionalis* | 12.3 | 43.5 |
| *L. v. meridionalis* | 13.0 | 43.4 |
| *L. v. meridionalis* | 12.9 | 43.3 |
| *L. v. meridionalis* | 14.6 | 41.5 |
| *L. v. meridionalis* | 10.2 | 44.0 |
| *L. v. meridionalis* | 10.2 | 44.0 |
| *L. v. meridionalis* | 10.5 | 42.9 |
| *L. v. meridionalis* | 10.5 | 43.0 |
| *L. v. meridionalis* | 10.6 | 43.3 |
| *L. v. meridionalis* | 10.7 | 43.2 |
| *L. v. meridionalis* | 10.9 | 43.3 |
| *L. v. meridionalis* | 11.0 | 42.8 |
| *L. v. meridionalis* | 11.1 | 42.6 |
| *L. v. meridionalis* | 11.1 | 42.6 |
| *L. v. meridionalis* | 11.0 | 43.3 |
| *L. v. meridionalis* | 11.2 | 42.6 |
| *L. v. meridionalis* | 11.1 | 42.7 |
| *L. v. meridionalis* | 11.1 | 42.9 |
| *L. v. meridionalis* | 11.1 | 43.2 |
| *L. v. meridionalis* | 11.1 | 43.3 |
| *L. v. meridionalis* | 11.2 | 43.0 |
| *L. v. meridionalis* | 11.3 | 43.2 |
| *L. v. meridionalis* | 11.3 | 43.3 |
| *L. v. meridionalis* | 11.5 | 42.5 |
| *L. v. meridionalis* | 11.5 | 43.2 |
| *L. v. meridionalis* | 10.3 | 43.6 |
| *L. v. meridionalis* | 10.3 | 43.7 |
| *L. v. meridionalis* | 10.3 | 43.9 |
| *L. v. meridionalis* | 10.3 | 43.8 |
| *L. v. meridionalis* | 10.4 | 43.4 |
| *L. v. meridionalis* | 10.4 | 43.6 |
| *L. v. meridionalis* | 10.5 | 43.6 |
| *L. v. meridionalis* | 10.4 | 43.8 |
| *L. v. meridionalis* | 10.6 | 43.8 |
| *L. v. meridionalis* | 10.7 | 43.7 |
| *L. v. meridionalis* | 10.7 | 43.8 |
| *L. v. meridionalis* | 10.7 | 43.7 |
| *L. v. meridionalis* | 10.9 | 43.8 |
| *L. v. meridionalis* | 10.8 | 43.9 |
| *L. v. meridionalis* | 10.8 | 43.4 |
| *L. v. meridionalis* | 10.9 | 43.5 |
| *L. v. meridionalis* | 10.9 | 43.8 |
| *L. v. meridionalis* | 11.0 | 43.9 |
| *L. v. meridionalis* | 11.1 | 43.4 |
| *L. v. meridionalis* | 11.1 | 43.6 |
| *L. v. meridionalis* | 10.9 | 43.8 |
| *L. v. meridionalis* | 11.1 | 43.6 |
| *L. v. meridionalis* | 11.3 | 43.8 |
| *L. v. meridionalis* | 11.2 | 43.8 |
| *L. v. meridionalis* | 11.1 | 43.9 |
| *L. v. meridionalis* | 11.2 | 44.0 |
| *L. v. meridionalis* | 11.3 | 43.4 |
| *L. v. meridionalis* | 11.3 | 43.6 |
| *L. v. meridionalis* | 11.3 | 43.9 |
| *L. v. meridionalis* | 11.2 | 44.0 |
| *L. v. meridionalis* | 11.2 | 44.0 |
| *L. v. meridionalis* | 11.5 | 43.6 |
| *L. v. meridionalis* | 11.4 | 43.8 |
| *L. v. meridionalis* | 11.4 | 44.1 |
| *L. v. meridionalis* | 11.5 | 43.3 |
| *L. v. meridionalis* | 11.6 | 42.9 |
| *L. v. meridionalis* | 11.8 | 42.9 |
| *L. v. meridionalis* | 11.8 | 42.9 |
| *L. v. meridionalis* | 11.9 | 43.3 |
| *L. v. meridionalis* | 11.6 | 43.4 |
| *L. v. meridionalis* | 11.6 | 43.6 |
| *L. v. meridionalis* | 11.5 | 43.8 |
| *L. v. meridionalis* | 11.5 | 43.8 |
| *L. v. meridionalis* | 11.6 | 43.5 |
| *L. v. meridionalis* | 11.6 | 43.6 |
| *L. v. meridionalis* | 11.7 | 43.8 |
| *L. v. meridionalis* | 11.9 | 43.5 |
| *L. v. meridionalis* | 11.7 | 43.5 |
| *L. v. meridionalis* | 11.8 | 43.8 |
| *L. v. meridionalis* | 12.0 | 43.6 |
| *L. v. meridionalis* | 11.9 | 43.7 |
| *L. v. meridionalis* | 11.8 | 43.8 |
| *L. v. meridionalis* | 11.9 | 43.8 |
| *L. v. meridionalis* | 12.0 | 43.6 |
| *L. v. meridionalis* | 12.1 | 43.6 |
| *L. v. meridionalis* | 12.2 | 43.7 |
| *L. v. meridionalis* | 10.5 | 43.8 |
| *L. v. meridionalis* | 12.2 | 43.7 |
