# Supplementary material for: Unraveling climate influences on the distribution of the parapatric newts Lissotriton vulgaris meridionalis and L. italicus
Source: Front Zool. 2017 Dec 12;14:55. doi: 10.1186/s12983-017-0239-4 (PMC5727953; doi:10.1186/s12983-017-0239-4)
Supplement: Supplementary file 2 — Correlation matrix. Correlation matrix built to select the non-correlated bioclimatic variables as predictors for the SDMs. Names of the variables with high values of autocorrelation (Pearson’s r > 0.85, in red, or r < −0.85, in brown) are highlighted in yellow (DOC 91 kb) [file 12983_2017_239_MOESM2_ESM.doc]

Additional file 2

|  |  | BIO1 | BIO2 | BIO3 | BIO4 | BIO5 | BIO6 | BIO7 | BIO8 | BIO9 | BIO10 | BIO11 | BIO12 | BIO13 | BIO14 | BIO15 | BIO16 | BIO17 | BIO18 | BIO19 | ALT | ASPECT | SLOPE |
| --- | --- | --- | --- | --- | --- | --- | --- | --- | --- | --- | --- | --- | --- | --- | --- | --- | --- | --- | --- | --- | --- | --- | --- |
| BIO1 | | 1.00 | 0.49 | 0.71 | -0.46 | 0.92 | 0.93 | 0.00 | 0.74 | 0.68 | 0.98 | 0.97 | -0.40 | -0.13 | -0.68 | 0.54 | -0.10 | -0.67 | -0.67 | 0.17 | -0.90 | -0.04 | -0.53 |
| BIO2 | | 0.49 | 1.00 | 0.88 | 0.05 | 0.74 | 0.22 | 0.72 | 0.34 | 0.36 | 0.54 | 0.40 | -0.06 | -0.02 | -0.12 | -0.03 | -0.05 | -0.04 | -0.12 | -0.02 | -0.62 | -0.01 | -0.41 |
| BIO3 | | 0.71 | 0.88 | 1.00 | -0.41 | 0.79 | 0.56 | 0.31 | 0.45 | 0.51 | 0.68 | 0.70 | -0.19 | 0.02 | -0.49 | 0.34 | 0.03 | -0.42 | -0.44 | 0.19 | -0.69 | -0.01 | -0.44 |
| BIO4 | | -0.46 | 0.05 | -0.41 | 1.00 | -0.17 | -0.70 | 0.72 | -0.23 | -0.36 | -0.29 | -0.65 | 0.17 | -0.18 | 0.76 | -0.78 | -0.24 | 0.75 | 0.64 | -0.51 | 0.19 | -0.01 | 0.11 |
| BIO5 | | 0.92 | 0.74 | 0.79 | -0.17 | 1.00 | 0.73 | 0.37 | 0.68 | 0.63 | 0.96 | 0.83 | -0.35 | -0.17 | -0.48 | 0.29 | -0.16 | -0.45 | -0.50 | 0.03 | -0.92 | -0.04 | -0.56 |
| BIO6 | | 0.93 | 0.22 | 0.56 | -0.70 | 0.73 | 1.00 | -0.36 | 0.63 | 0.65 | 0.86 | 0.98 | -0.45 | -0.12 | -0.82 | 0.70 | -0.06 | -0.83 | -0.80 | 0.28 | -0.71 | -0.03 | -0.41 |
| BIO7 | | 0.00 | 0.72 | 0.31 | 0.72 | 0.37 | -0.36 | 1.00 | 0.06 | -0.02 | 0.14 | -0.19 | 0.13 | -0.08 | 0.46 | -0.56 | -0.15 | 0.53 | 0.40 | -0.34 | -0.29 | -0.01 | -0.20 |
| BIO8 | | 0.74 | 0.34 | 0.45 | -0.23 | 0.68 | 0.63 | 0.06 | 1.00 | 0.25 | 0.74 | 0.69 | -0.30 | -0.17 | -0.35 | 0.30 | -0.14 | -0.36 | -0.33 | -0.09 | -0.73 | -0.04 | -0.44 |
| BIO9 | | 0.68 | 0.36 | 0.51 | -0.36 | 0.63 | 0.65 | -0.02 | 0.25 | 1.00 | 0.66 | 0.67 | -0.29 | -0.08 | -0.56 | 0.37 | -0.06 | -0.50 | -0.61 | 0.24 | -0.61 | -0.02 | -0.33 |
| BIO10 | | 0.98 | 0.54 | 0.68 | -0.29 | 0.96 | 0.86 | 0.14 | 0.74 | 0.66 | 1.00 | 0.92 | -0.41 | -0.19 | -0.58 | 0.41 | -0.16 | -0.57 | -0.60 | 0.07 | -0.93 | -0.05 | -0.55 |
| BIO11 | | 0.97 | 0.40 | 0.70 | -0.65 | 0.83 | 0.98 | -0.19 | 0.69 | 0.67 | 0.92 | 1.00 | -0.41 | -0.09 | -0.78 | 0.66 | -0.04 | -0.77 | -0.75 | 0.26 | -0.81 | -0.04 | -0.48 |
| BIO12 | | -0.40 | -0.06 | -0.19 | 0.17 | -0.35 | -0.45 | 0.13 | -0.30 | -0.29 | -0.41 | -0.41 | 1.00 | 0.89 | 0.47 | -0.03 | 0.87 | 0.58 | 0.68 | 0.60 | 0.27 | 0.11 | 0.31 |
| BIO13 | | -0.13 | -0.02 | 0.02 | -0.18 | -0.17 | -0.12 | -0.08 | -0.17 | -0.08 | -0.19 | -0.09 | 0.89 | 1.00 | 0.06 | 0.39 | 0.99 | 0.17 | 0.33 | 0.82 | 0.12 | 0.12 | 0.22 |
| BIO14 | | -0.68 | -0.12 | -0.49 | 0.76 | -0.48 | -0.82 | 0.46 | -0.35 | -0.56 | -0.58 | -0.78 | 0.47 | 0.06 | 1.00 | -0.83 | 0.01 | 0.96 | 0.90 | -0.32 | 0.42 | 0.01 | 0.29 |
| BIO15 | | 0.54 | -0.03 | 0.34 | -0.78 | 0.29 | 0.70 | -0.56 | 0.30 | 0.37 | 0.41 | 0.66 | -0.03 | 0.39 | -0.83 | 1.00 | 0.45 | -0.81 | -0.66 | 0.65 | -0.26 | 0.03 | -0.11 |
| BIO16 | | -0.10 | -0.05 | 0.03 | -0.24 | -0.16 | -0.06 | -0.15 | -0.14 | -0.06 | -0.16 | -0.04 | 0.87 | 0.99 | 0.01 | 0.45 | 1.00 | 0.11 | 0.28 | 0.87 | 0.12 | 0.11 | 0.22 |
| BIO17 | | -0.67 | -0.04 | -0.42 | 0.75 | -0.45 | -0.83 | 0.53 | -0.36 | -0.50 | -0.57 | -0.77 | 0.58 | 0.17 | 0.96 | -0.81 | 0.11 | 1.00 | 0.93 | -0.24 | 0.38 | 0.04 | 0.27 |
| BIO18 | | -0.67 | -0.12 | -0.44 | 0.64 | -0.50 | -0.80 | 0.40 | -0.33 | -0.61 | -0.60 | -0.75 | 0.68 | 0.33 | 0.90 | -0.66 | 0.28 | 0.93 | 1.00 | -0.11 | 0.42 | 0.06 | 0.32 |
| BIO19 | | 0.17 | -0.02 | 0.19 | -0.51 | 0.03 | 0.28 | -0.34 | -0.09 | 0.24 | 0.07 | 0.26 | 0.60 | 0.82 | -0.32 | 0.65 | 0.87 | -0.24 | -0.11 | 1.00 | -0.02 | 0.08 | 0.12 |
| ALT | | -0.90 | -0.62 | -0.69 | 0.19 | -0.92 | -0.71 | -0.29 | -0.73 | -0.61 | -0.93 | -0.81 | 0.27 | 0.12 | 0.42 | -0.26 | 0.12 | 0.38 | 0.42 | -0.02 | 1.00 | 0.03 | 0.59 |
| ASPECT | | -0.04 | -0.01 | -0.01 | -0.01 | -0.04 | -0.03 | -0.01 | -0.04 | -0.02 | -0.05 | -0.04 | 0.11 | 0.12 | 0.01 | 0.03 | 0.11 | 0.04 | 0.06 | 0.08 | 0.03 | 1.00 | 0.04 |
| SLOPE | | -0.53 | -0.41 | -0.44 | 0.11 | -0.56 | -0.41 | -0.20 | -0.44 | -0.33 | -0.55 | -0.48 | 0.31 | 0.22 | 0.29 | -0.11 | 0.22 | 0.27 | 0.32 | 0.12 | 0.59 | 0.04 | 1.00 |
