# Supplementary material for: Unraveling climate influences on the distribution of the parapatric newts Lissotriton vulgaris meridionalis and L. italicus
Source: Front Zool. 2017 Dec 12;14:55. doi: 10.1186/s12983-017-0239-4 (PMC5727953; doi:10.1186/s12983-017-0239-4)
Supplement: Supplementary file 3 — WorldClim bioclimatic variables. Codes and explication, as reported in the WorldClim website, of the nineteen bioclimatic variables considered as candidate predictors (DOC 23 kb) [file 12983_2017_239_MOESM3_ESM.doc]

**Additional file 3**

BIO1 = Annual Mean Temperature

BIO2 = Mean Diurnal Range (Mean of monthly (max temp – min temp))

BIO3 = Isothermality (BIO2/BIO7)*100

BIO4 = Temperature Seasonality (standard deviation*100)

BIO5 = Max Temperature of Warmest Month

BIO6 = Min Temperature of Coldest Month

BIO7 = Temperature Annual Range (BIO5-BIO6)

BIO8 = Mean Temperature of Wettest Quarter

BIO9 = Mean Temperature of Driest Quarter

BIO10 = Mean Temperature of Warmest Quarter

BIO11 = Mean Temperature of Coldest Quarter

BIO12 = Annual Precipitation

BIO13 = Precipitation of Wettest Month

BIO14 = Precipitation of Driest Month

BIO15 = Precipitation Seasonality (Coefficient of Variation)

BIO16 = Precipitation of Wettest Quarter

BIO17 = Precipitation of Driest Quarter

BIO18 = Precipitation of Warmest Quarter

BIO19 = Precipitation of Coldest Quarter
