# Supplementary material for: Unraveling climate influences on the distribution of the parapatric newts Lissotriton vulgaris meridionalis and L. italicus
Source: Front Zool. 2017 Dec 12;14:55. doi: 10.1186/s12983-017-0239-4 (PMC5727953; doi:10.1186/s12983-017-0239-4)

Additional file 4

| *Lissotriton italicus* | | *Lissotriton vulgaris meridionalis* | |
| --- | --- | --- | --- |
| Variable | Relative contribution (%) | Variable | Relative contribution (%) |
| BIO19 | 31.0 | BIO17 | 41.1 |
| BIO17 | 18.1 | BIO12 | 13.1 |
| BIO15 | 9.3 | BIO2 | 12.2 |
| BIO9 | 8.4 | BIO7 | 7.7 |
| BIO7 | 8.0 | BIO6 | 7.3 |
| BIO2 | 7.2 | BIO15 | 6.1 |


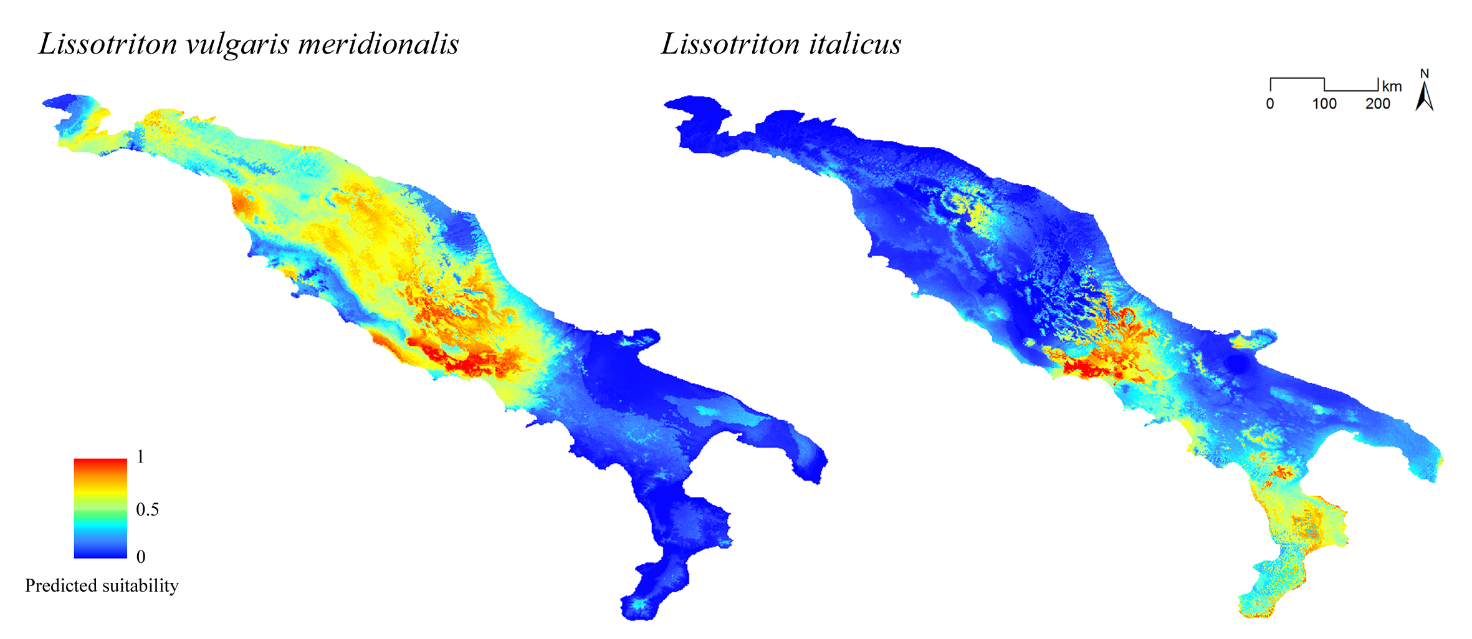

Supplement: Supplementary file 4 — Highly contributing variables and predicted suitability maps from the Maxent models. For each Lissotriton species are reported the relative contributions of the six most influential predictors and the maps of predicted suitability, as results from the corresponding Maxent models built under the current climatic conditions (DOC 455 kb) [file 12983_2017_239_MOESM4_ESM.doc]
