# Supplementary material for: Unraveling climate influences on the distribution of the parapatric newts Lissotriton vulgaris meridionalis and L. italicus
Source: Front Zool. 2017 Dec 12;14:55. doi: 10.1186/s12983-017-0239-4 (PMC5727953; doi:10.1186/s12983-017-0239-4)

Additional file 5

| Species | Scenario | Area of extrapolation (Km^2^) (MESS < -20) | (Area of extrapolation / total study area) * 100 |
| --- | --- | --- | --- |
| *L. italicus* | Current | 36.14 | 0.02 |
| *L. italicus* | MOL_MIROC | 7535.04 | 3.77 |
| *L. italicus* | MOL_CCSM4 | 15405.70 | 7.70 |
| *L. italicus* | LGM_MIROC | 124446.96 | 62.24 |
| *L. italicus* | LGM_CCSM4 | 54345.94 | 27.18 |
| *L. v. meridionalis* | Current | 12.91 | 0.01 |
| *L. v. meridionalis* | MOL_MIROC | 3795.06 | 1.90 |
| *L. v. meridionalis* | MOL_CCSM4 | 7387.89 | 3.69 |
| *L. v. meridionalis* | LGM_MIROC | 115233.83 | 57.63 |
| *L. v. meridionalis* | LGM_CCSM4 | 46576.82 | 23.29 |


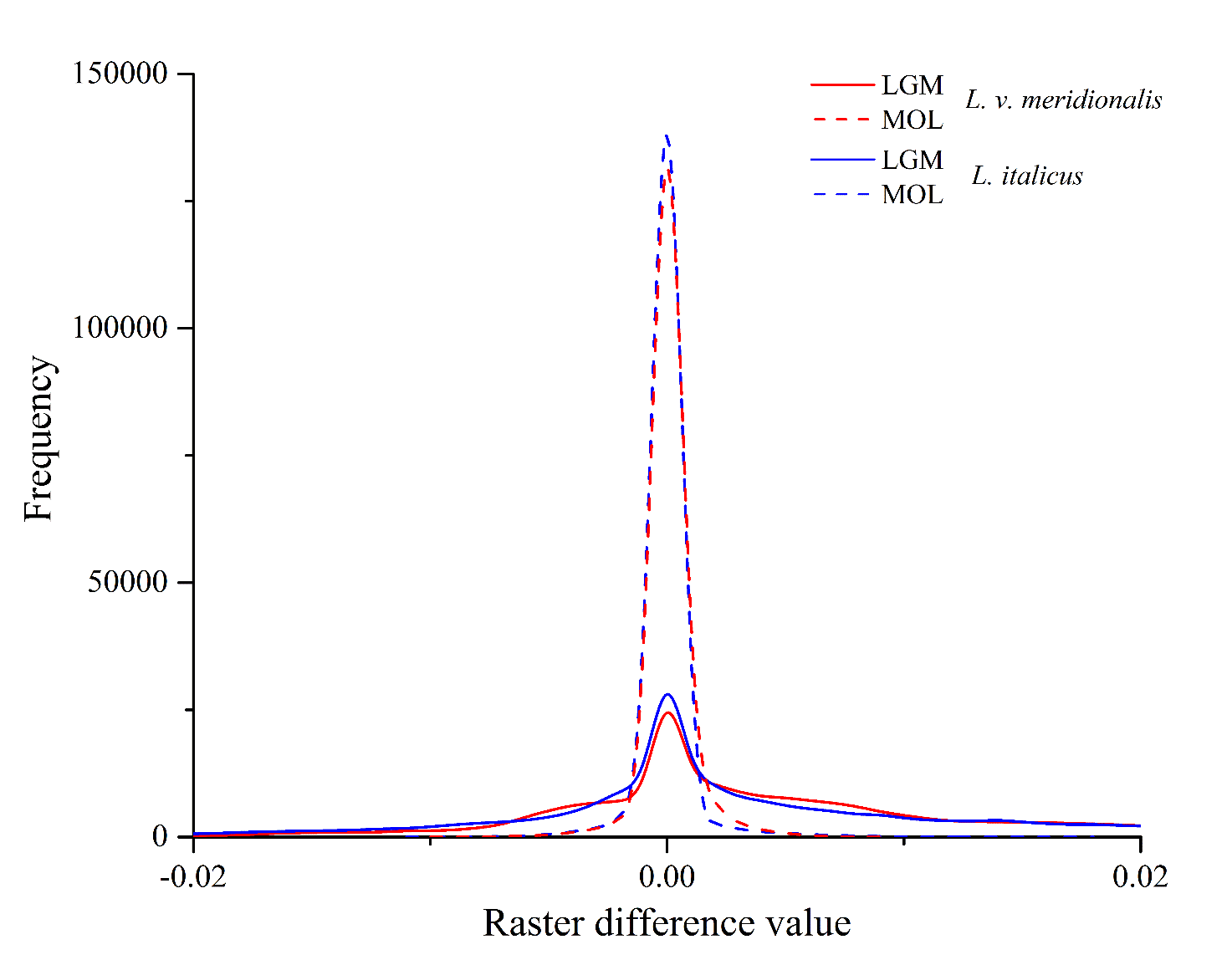

Supplement: Supplementary file 5 — Extent of model extrapolation and differences in model projections’ averages. The table shows the percent extent of the study area interested by a certain degree of extrapolation (MES < −20) for each combination of species-temporal scenario-GCM; the figure instead illustrates the differences between the raster suitability maps resulting from the weighted and simple average of the SDMs’ projections considering the MIROC-ESM and the CCSM4 paleoclimatic reconstructions (DOC 68 kb) [file 12983_2017_239_MOESM5_ESM.doc]
